# Autologous transplantation of thecal stem cells restores ovarian function in nonhuman primates

**DOI:** 10.1038/s41421-021-00291-0

**Published:** 2021-08-31

**Authors:** Hong Chen, Kai Xia, Weijun Huang, Huijian Li, Chao Wang, Yuanchen Ma, Jianhui Chen, Peng Luo, Shuwei Zheng, Jiancheng Wang, Yi Wang, Lin Dong, Zhipeng Tan, Xingqiang Lai, Frank Fuxiang Mao, Weiqiang Li, Xiaoyan Liang, Tao Wang, Andy Peng Xiang, Qiong Ke

**Affiliations:** 1 Center for Stem Cell Biology and Tissue Engineering, Key Laboratory for Stem Cells and Tissue Engineering, Ministry of Education, Sun Yat-sen University, Guangzhou, Guangdong China; 2 Department of Genetics and Cell Biology, Zhongshan School of Medicine, Sun Yat-sen University, Guangzhou, Guangdong China; 3 Center for Reproductive Medicine, The Sixth Affiliated Hospital, Sun Yat-sen University, Guangzhou, Guangdong China; 4 Department of Andrology, The First Affiliated Hospital, Sun Yat-sen University, Guangzhou, Guangdong China; 5 Scientific Research Center, The Seventh Affiliated Hospital, Sun Yat-sen University, Shenzhen, Guangdong China; 6 Cardiovascular Department, The Eighth Affiliated Hospital, Sun Yat-sen University, Shenzhen, Guangdong China; 7 State Key Laboratory of Ophthalmology, Zhong Shan Ophthalmic Center, Sun Yat-sen University, Guangzhou, Guangdong China; 8 Department of Biochemistry, Zhongshan School of Medicine, Sun Yat-sen University, Guangzhou, Guangdong China

**Keywords:** Mesenchymal stem cells, Regeneration

## Abstract

Premature ovarian insufficiency (POI) is defined as the loss of ovarian activity under the age of 40. Theca cells (TCs) play a vital role during folliculogenesis and TCs dysfunction participate in the pathogenesis of POI. Therefore, transplantation of thecal stem cells (TSCs), which are capable of self-renewal and differentiation into mature TCs, may provide a new strategy for treating POI. To investigate the feasibility, safety, and efficacy of TSCs transplantation in clinically relevant non-human primate (NHP) models, we isolate TSCs from cynomolgus monkeys, and these cells are confirmed to expand continuously and show potential to differentiate into mature TCs. In addition, engraftment of autologous TSCs into POI monkeys significantly improves hormone levels, rescues the follicle development, promotes the quality of oocytes and boosts oocyte maturation/fertilization rate. Taken together, these results for the first time suggest that autologous TSCs can ameliorate POI symptoms in primate models and shed new light on developing stem cell therapy for POI.

## Introduction

Premature ovarian insufficiency (POI), a syndrome defined by hypergonadotropic hypoestrogenism, causes amenorrhea and loss of ovarian activity and affects approximately 1% of women before the age of 40^1,2^. The gold-standard diagnosis is based on the change in the levels of the main ovarian hormones and the decrease in the number of follicles^3^. Endocrine, genetic, metabolic, infectious, and iatrogenic factors are implicated in this physiological or pathophysiological reproductive aging^4,5^. Hormone replacement therapy (HRT) has been used for the treatment of patients with POI, but HRT increases the risk of blood clots and breast cancer^6,7^. More importantly, clinical investigations have demonstrated that HRT offers little or no benefit in improving fertility^8^.

In recent studies, cell therapy has been considered a potential therapeutic alternative to traditional treatments of POI^9^. A variety of stem cells have been used to restore ovarian failure in mice, such as mesenchymal stem cells, extraembryonic stem cells, human amniotic fluid stem cells, human menstrual blood stem cells, and human amniotic epithelial cells^10–14^. Most of the studies have shown the normalization of hormonal levels, restoration of mature follicle production, and recovery of the menstrual cycle. However, the therapeutic effects of those treatments are still compromised given that stem cell therapy has not restored reproductive outcomes^10,15^. On the other hand, Besikcioglu et al. demonstrated that the transplantation of ovarian stromal stem cells (OSSCs) can be more effective than bone marrow mesenchymal stem cells (BMMSCs) for follicle maturation after chemotherapy in rats^16^. It seems that tissue resident stem cells are more suitable for their original tissue repair.

As the ovary resident somatic cells, theca cells (TCs) provide structural support for growing follicle and androgen substrates for estrogen production^17^. Midhun Soman et al. reported that women suffering from premature ovarian failure (POF) have lower androstenedione (A2) than controls, indicating that TCs dysfunction occurs in POF^18^. Notably, Chendi Zhu et al. revealed that insulin-like 3 (INSL3), produced by theca interna cells, continuously decreased with the progress of ovarian insufficiency, which indicates that circulating INSL3 could serve as a promising theca-cell biomarker for POI^19^. Conversely, TCs hyperproliferation occurs in polycystic ovary syndrome (PCOS), which is consistent with obvious follicular development, especially early antral follicle count (AFC), highlighting that TCs are indispensable for the growth and development of ovary follicles^20^. Thus, restoration of TCs function may provide a new strategy for treating POI.

TCs arise from thecal stem cells (TSCs), which are capable of self-renewal and differentiation into mature TCs. Arata Honda et al. reported that putative thecal stem cells exist in neonatal ovaries, differentiate into mature TCs and produce androstenedione^21^. After that, TSCs have been successfully isolated from pig, sheep, and humans, but the in vivo reparative function of TSCs transplantation has not been evaluated^22–24^.

Nonhuman primates (NHPs) are the most appropriate animal model for studying ovarian aging and dysfunction^25^. More importantly, cynomolgus monkeys (*Macaca fascicularis*) share similar genetic and physiological features with human females, especially the reproductive cycle and steroidogenesis^25,26^. Our group had experience in a gene-editing model in NHPs for human diseases^27,28^ and observed that consecutive superovulated oocyte donors resembling the phenotypes of POI as reported^29^, which might provide a suitable model of POI to address the challenging translational aspects of cellular therapy and bridge the translational gap from small-animal models to humans.

Here, we first isolated and characterized cynomolgus monkey TSCs and demonstrated that autologous TSCs transplantation partially relieved the relevant symptoms of POI in a primate model. These findings support the need for large-scale studies with long-term follow-up to assess the translational feasibility of autologous TSCs for POI patients.

## Results

### Isolation, characterization, and long-term culture of TSCs from ovary biopsies of cynomolgus monkeys

**Fig. 1:**
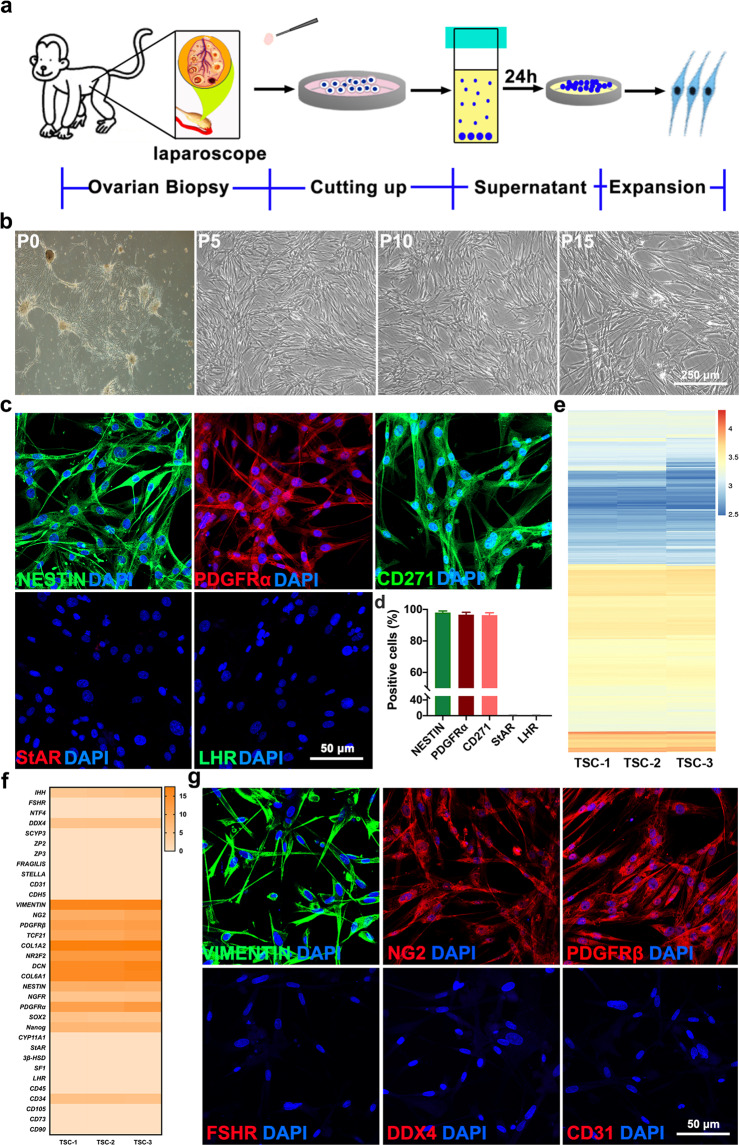
Isolation and characterization of TSCs from the ovaries of cynomolgus monkeys with POI. **a** Schematic of the experimental procedure used to obtain primary thecal stem cells (TSCs). **b** Phase-contrast micrographs of TSCs at primary culture passage 0 (P0), passage 5 (P5), passage 10 (P10), and passage 15 (P15). **c** Cultured TSCs expressed NESTIN, CD271, and PDGFRα, but not StAR or LHR. Nuclei were counterstained with DAPI (blue). **d** Statistical analysis of expression markers of TSCs and cell replicates (*n* = 3). **e** Heatmap showed expression of gene for the TSCs (*n* = 3). **f** Heatmap showed expression of marker genes for the major cell types in the TSCs (*n* = 3). **g** Immunostaining showed the TSCs expressed markers about interstitial cells, but not granulose cells, oocytes, and endothelial cells. Data are expressed as the means ± SEM.

For the purpose of autologous TSCs transplantation for treatment, cell culture from a small amount of ovary would minimize the damage of the living organism. Here, we introduced an enzyme-free method to obtain a cell suspension from a small piece of ovarian biopsies (Fig. 1a). Observation after 4 weeks of sampling revealed no effects on weight, ejection fraction (LVEF), uterine size, ovarian volume, hormone level, or inflammatory reaction (Supplementary Fig. S1 and Table S1). Two days after plating, cells attached to the surface of the tissue-culture plate with a fibroblast-like spindle-shaped morphology and reached approximately 10% confluence. Approximately 2 weeks later, those cells reached 70%–80% confluence and exhibited proliferative ability in the TSCs culture medium system (Fig. 1b).

Arata Honda et al. demonstrated that the TCs and Leydig cells (LCs) shared similar characteristics and might originate from a common progenitor population^21,30^. To investigate whether TSCs could express the specific markers of stem Leydig cells (SLCs) that others and our previous studies reported^31–34^, immunofluorescence staining was performed. We found that TSCs highly expressed NESTIN (98.00 ± 0.58%), platelet-derived growth factor receptor-α (PDGFRα) (96.67 ± 0.88%) and CD271 (P75 neurotrophin receptor) (96.33 ± 0.88%), but did not express the TCs markers, such as steroidogenic acute regulatory protein (StAR) and LHR, at P5 (Fig. 1c, d). When detected by flow cytometry, the cultured cells expressed PDGFRα constantly at different passages (P3, P5, P10, and P15) (Supplementary Fig. S2a–c).

To further determine the characteristics of these cells, the gene expression pattern of cells was observed by RNA sequencing (RNA-seq) (Fig. 1e). Consistent with the results of immunohistochemistry, it clearly expressed stemness characteristics such as *NESTIN, PDGFRα,* and *NGFR*, but not express the markers of mature TCs, such as *LHR, StAR* (Fig. 1f and Supplementary Fig. S2d). These cells also expressed mesenchymal linage markers *VIMENTIN, NG2, PDGFRβ* but not *CD90, CD73, CD105*, displaying the characteristics of mesenchymal cells. More importantly, this group of cells does not express the characteristic markers of oocytes (*SCYP3, ZP3*), granulosa cells (*NTF4, FSHR*), and endothelial cells (*CD31, CDH5*) (Fig. 1f). This is consistent with the results of immunohistochemistry (Fig. 1g).

Additionally, the cultured cells of different passages did not show any remarkable difference in passage proliferation rate or population doubling time, indicating that these cells exhibited self-renewal capacity in vitro (Supplementary Fig. S2e, f). Moreover, we did not observe any chromosomal abnormalities of TSCs at P5 or P15 (Supplementary Fig. S2g). Although the TSCs (P5 or P10) were inoculated subcutaneously into NCG mice at 8 weeks old, we failed to observe any tumor formation after 3 months. In contrast, mice injected with testis cancer cells exhibited efficient tumor formation (100%) (Supplementary Fig. S2h).

All of these results indicate that the ovarian biopsy explant culture for derivation of primary TSCs is safe and feasible, and that the cells exhibit self-renewal potential.

### Multilineage differentiation capacity of TSCs in vitro

**Fig. 2:**
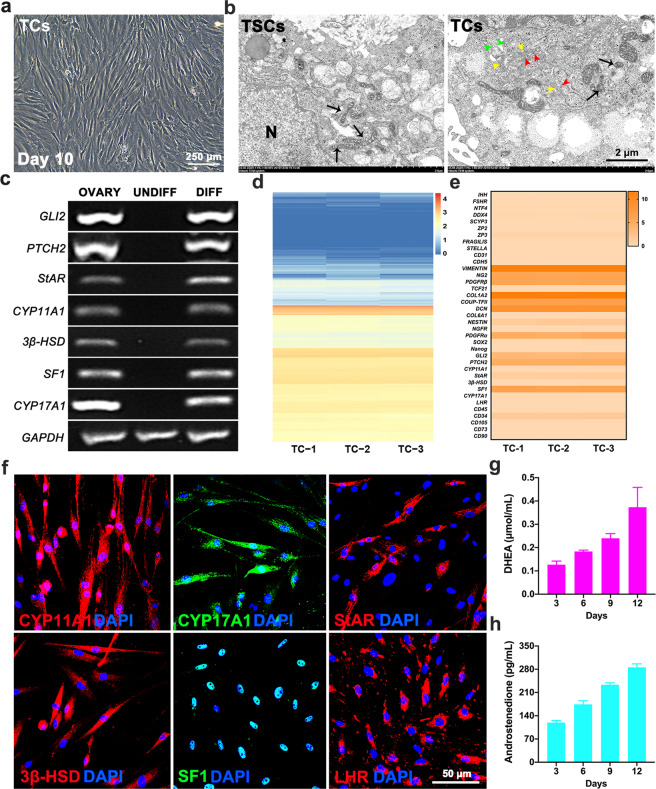
Multilineage differentiation capacity of TSCs in vitro. **a** Phase-contrast micrographs of TSCs after 10 days of differentiation. **b** The TSCs and TCs organelles were observed under electron microscope. mitochondria with lamellae or typical tubular cristae (black arrow), smooth ER (red arrow), Golgi apparatuses (yellow arrow), vacuoles (green arrow), N nucleus. **c** RT-PCR analysis confirmed that the expression of the TCs lineage-specific markers was increased in differentiated cells (DIFF) compared with undifferentiated controls (UNDIFF), in which the markers were undetectable. **d** Heatmap showed expression of gene for the TCs (*n* = 3). **e** Heatmap showed expression of marker genes for the major cell types in the TCs (*n* = 3). **f** After differentiation for 12 days, immunofluorescence staining showed that the TSCs clearly expressed the TCs lineage-specific markers, CYP11A1, CYP17A1, StAR, 3β-HSD, SF-1, and LHR. **g** Dehydroepiandrosterone production progressively increased during culture in differentiation-inducing time. **h** Androstenedione production progressively increased during culture in differentiation-inducing time (*n* = 3).

To investigate the differentiation potential of TSCs, we treated the cells with differentiated culture medium and cocultured them with GCs as Arata Honda et al. reported^21^. We observed a broad range of cell morphology as assessed by bright-field microscopy (Fig. 2a). Electron microscopic observations revealed that differentiated cells exhibited more steroid-producing cell features. Their cytoplasm was rich in smooth endoplasmic reticulum, Golgi apparatuses, small vacuoles, and numerous mitochondria with tubular cristae (Fig. 2b). To further confirm that the differentiated cells were TCs, RT-PCR analysis were performed and showed that TSCs-derived cells expressed mature TCs markers, such as GLI Family Zinc Finger 2 (*GLI2*), protein patched homolog 2 (*PTCH2*), *StAR*, cytochrome p450 family 11 subfamily A member 1 (*CYP11A1*), 3-beta-hydroxysteroid dehydrogenase (*3β-HSD*), steroidogenic factor-1 (*SF1*), and cytochrome p450 family 17 subfamily A member 1 (*CYP17A1*), after 12 days of induction as reported^21,22^ (Fig. 2c). Further, to examine the transcript profile of the TCs genome, RNA-Seq were performed and those cell lines showed the similar expression patterns (Fig. 2d). The expression of mature TCs related genes was significantly increased, while the stemness-related genes were decreased (Fig. 2e). Consistent with this, the results of immunohistochemistry showed that these cells can express TCs-related steroidogenic enzymes, including CYP11A1, CYP17A1, StAR, 3β-HSD, SF1, and LHR in protein level (Fig. 2f). After long-term culture in vitro, TSCs retained their potential to differentiate into TCs (Supplementary Fig. S3a). Additionally, we found that the concentrations of dehydroepiandrosterone (DHEA) and A2 gradually increased over time following the TCs differentiation in the cell culture supernatants (Fig. 2g, h). The difference was significant on day 12 for DHEA (0.37 ± 0.05 μmol/mL) and A2 (285.70 ± 6.02 pg/mL) compared with day 3 (0.13 ± 0.01 μmol/mL and 119.16 ± 3.52 pg/mL, respectively). In addition, these TSCs also had the ability to differentiate into osteoblast, adipocyte, and chondrocyte lineages (Supplementary Fig. S3b–g). These findings demonstrate that TSCs have the multiple differentiation potentials.

### Establishment of POI models with the evaluation of relevant morphological and histopathological parameters

Nie et al. reported that consecutive superovulation could be used to establish a mouse model of ovarian aging^29^. We found that the number and quality of retrieved oocytes significantly decreased after several rounds of controlled ovarian stimulation (COS) in some of the oocyte donor monkeys used in our previous study^27,28^ (Supplementary Fig. S4a–d). When monitoring hormone levels, the results indicated that some of the monkeys had the similar hormone changes in patients with POI^35^. Serum anti-Mullerian hormone (AMH) levels in those monkeys significantly decreased from 14.32 ± 1.05 to 5.82 ± 0.24 ng/mL, whereas estradiol (E2) levels decreased from 325.80 ± 54.21 to 173.48 ± 21.80 pg/mL (Supplementary Fig. S4e, f). The follicle stimulating hormone (FSH) level was increased 2-fold from 4.80 ± 0.41 to 9.93 ± 0.24 mIU/mL (Supplementary Fig. S4g). More importantly, these female monkeys also suffered from menstrual cycle disorders, earlier menopause and infertility (data not shown). We also found that the number of follicles at different stages was significantly reduced in POI monkeys, especially the secondary follicles (43 ± 2.5 follicles/side in the control group versus 11 ± 0.8 follicles/side in the POI group) (Supplementary Fig. S4h–l). Therefore, these results indicate that these monkeys may represent a suitable animal model of POI.

### Autologous transplantation of TSCs improves hormone levels in POI monkeys

**Fig. 3:**
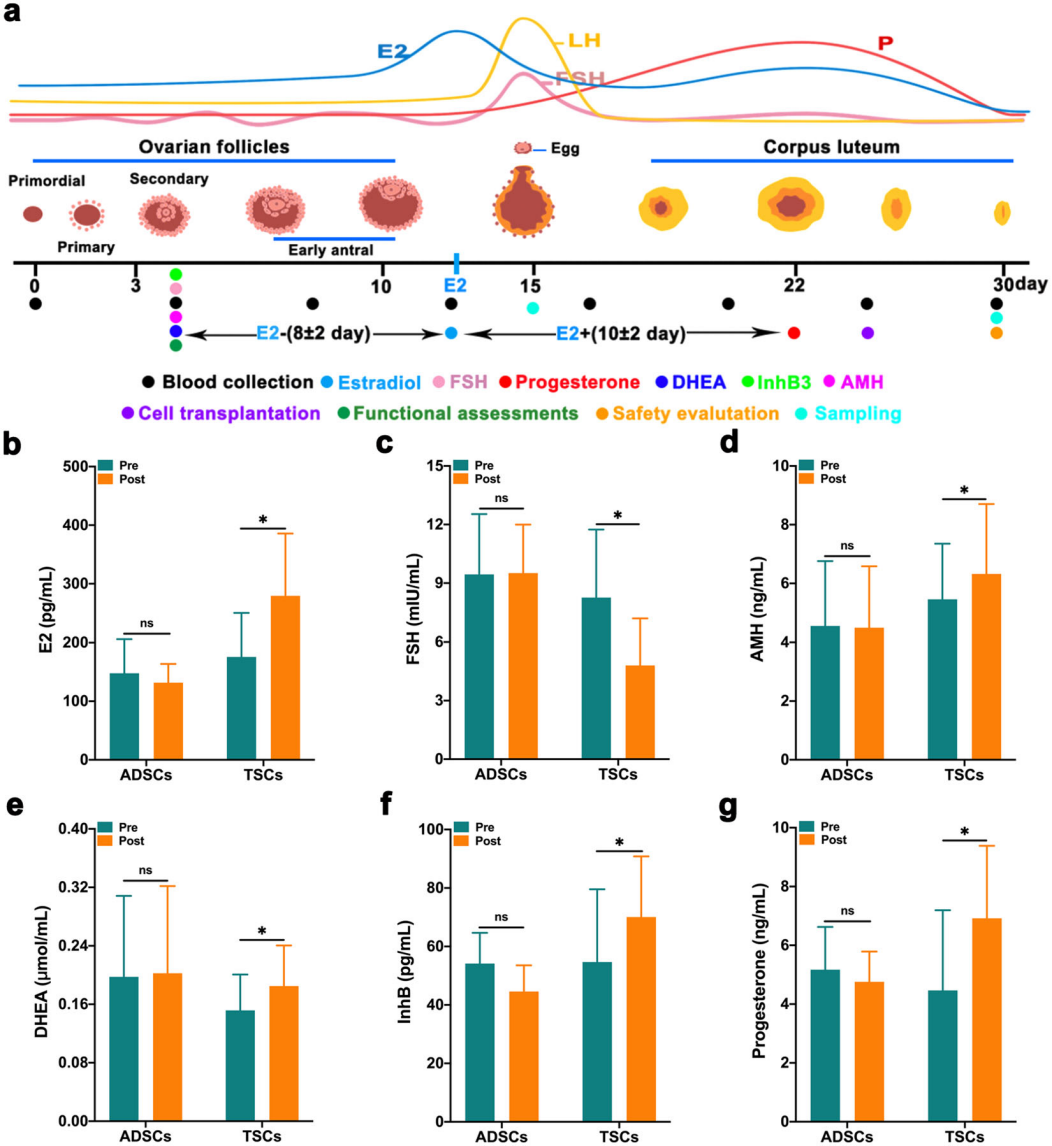
TSCs transplantation improves hormone levels in monkeys with POI. **a** Schematic of the experimental procedure used for cell transplantation, functional and safety assessment. The black dots represent the point at which the blood was collected, and the dots in different colors represent the time selected by different functional assessments, which were all calculated based on the peak E2 time. The dark green dots represent functional assessments including ovarian, uterine and heart function. **b**–**g** Estrogen peak (**b**), follicle stimulating hormone (**c**), anti-Mullerian Hormone (**d**), dehydroepiandrosterone (**e**), InhB (**f**), and progesterone peak (**g**). The ADSCs group represents the monkeys with POI who received adipose-derived mesenchymal stem cells transplantation (ADSCs = POI + ADSCs, *n* = 4); the TSCs group represents the monkeys with POI who received thecal stem cells transplantation (TSCs = POI + TSCs, *n* = 6). Data are expressed as the means ± SEM and were assessed using *t*-test; *P < 0.05, ns not significant.

To determine the reparative function of TSCs in vivo, we performed autologous transplantation experiments to investigate whether TSCs could improve the ovarian function in a POI monkey model. Autologous adipose-derived mesenchymal stem cells (ADSCs) were used as control and the characterization of ADSCs were showed in Supplementary Fig. S5a, b. Twelve monkeys that had conditions indicative of AMH (<5.11 ± 0.62 ng/mL), E2 (<164.50 ± 21.20 pg/mL), and FSH (>8.74 ± 1.01 mIU/mL) were enrolled and randomly divided into two groups. One group received ADSCs transplantation (*n* = 6), whereas the other group received TSCs transplantation (*n* = 6) (Supplementary Table S2). Cell transplantation was performed on the day during the corpus luteum, and the ovarian function was evaluated as shown in Fig. 3a. Excitingly, the peak of E2 significantly increased (175.00 ± 30.72 pg/mL versus 279.87 ± 43.24 pg/mL) in the TSCs group, and the FSH level was significantly declined from 8.28 ± 1.42 to 4.79 ± 0.99 mIU/mL in the next menstrual cycle (Fig. 3b, c). The other hormone levels also increased, including AMH (5.47 ± 0.77 to 6.32 ± 0.97 ng/mL), DHEA (0.15 ± 0.02 to 0.19 ± 0.02 µmol/mL), InhB (54.67 ± 10.15 to 70.08 ± 8.47 pg/mL), and progesterone (4.47 ± 1.11 to 6.92 ± 1.01 ng/mL) (Fig. 3d–g). In contrast, there were no remarkable changes in serum hormone levels before and after transplantation in the ADSCs group. More importantly, we observed that the improvement in serum E2 could last for approximately three menstrual cycles (Supplementary Fig. S5c). Both TSCs and ADSCs transplantation had no discernible ill effects in the monkey models (data not shown). In summary, these results demonstrate that autologous transplantation of TSCs can effectively improve ovarian hormone levels in POI monkey models.

### Autologous transplantation of TSCs promotes the development of ovarian follicles in POI monkeys

**Fig. 4:**
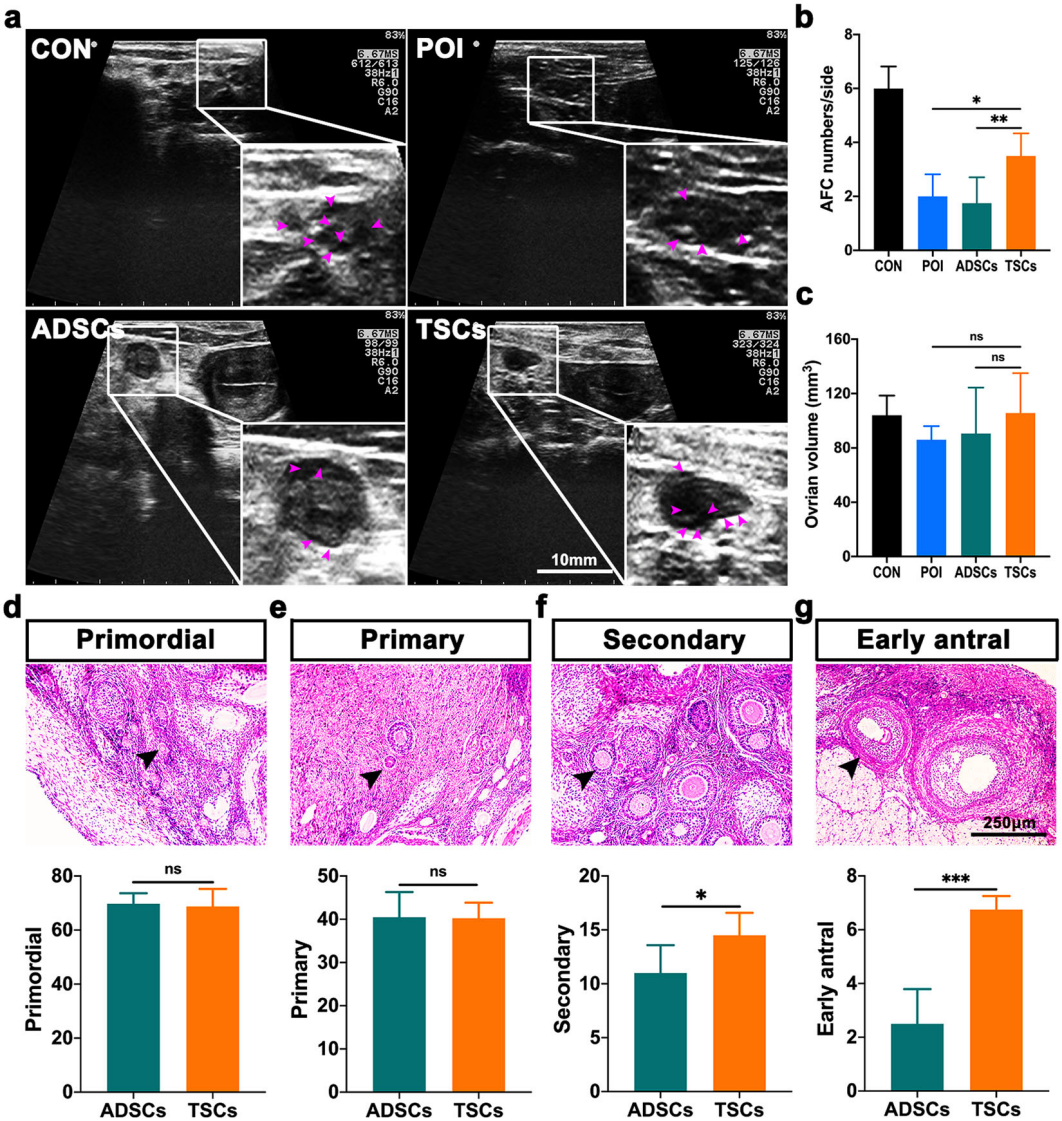
TSCs transplantation was beneficial to the development of AFC. **a** B-ultrasound observation of ovarian development and statistics of early antral count (AFC) (purple arrow refers to the AFC, diameter ≥ 2 mm) after TSCs transplantation. **b** Statistics of the number of AFCs. **c** Statistics of the ovarian volume. **d** The number of primordial follicles (indicated by the black arrow). **e** The number of primary follicles (indicated by the black arrow). **f** The number of secondary follicles (indicated by the black arrow). **g** The number of early antral follicles (indicated by the black arrow). (Compare to control, *n* = 4). The CON group represents the normal wild-type monkeys (*n* = 4), the POI group represents the monkeys with premature ovarian failure (*n* = 4), the ADSCs group represents the monkeys with POI that received adipose-derived mesenchymal stem cells transplantation (ADSCs = POI + ADSCs, *n* = 4); and the TSCs group represents the monkeys with POI that received thecal stem cells transplantation (TSCs = POI + TSCs, *n* = 4). Data are expressed as the means ± SEM and significance were determined by one-way ANOVA or *t*-test. *P < 0.05, **P < 0.01, ns not significant; *P < 0.05, **P < 0.01, ***P < 0.001, ns not significant.

AFC is an important indicator of ovarian function evaluation, and a decrease in AFC is a typical feature in patients with POI^36,37^. To assess whether the TSCs transplantation could promote follicle development, we counted the number of AFC on the 15th day of the next menstruation under ultrasound guidance. After TSCs transplantation, the number of antral follicles in the POI monkeys was increased distinctly (1 ± 0.2 follicles/side versus 4 ± 0.3 follicles/side) (Fig. 4a, b), and persisted for up to three menstrual cycles, whereas no changes were noted in the ADSCs group (Supplementary Fig. S5d). The volume of ovaries and uterus showed no changes in either the TSCs group or ADSCs group (Fig. 4c and Supplementary Fig. S5e).

To further investigate the number of follicles at different stages, ovaries were obtained and fixed 20 days (before ovulation) and 30 days (at the luteal phase) after cell transplantation. Hematoxylin-eosin staining showed that the total number of follicles was not remarkably different between the TSCs group and the ADSCs group (124 ± 3.4 vs 130 ± 4.2) (Supplementary Fig. S5f, g). Although the number of primordial follicles and primary follicles did not seem to be affected in either group, the number of the secondary follicles (11 ± 1.3 in the ADSCs group vs 15 ± 1.0 in the TSCs group) and the early antral follicles (3 ± 0.6 in the ADSCs group versus 7 ± 0.3 in the TSCs group) were significantly improved in the TSCs group (Fig. 4d–g). All of these results prove that autologous TSCs transplantation could promote the development of ovarian follicles, especially AFC.

### TSCs differentiate into TCs and participate in follicular development in vivo

**Fig. 5:**
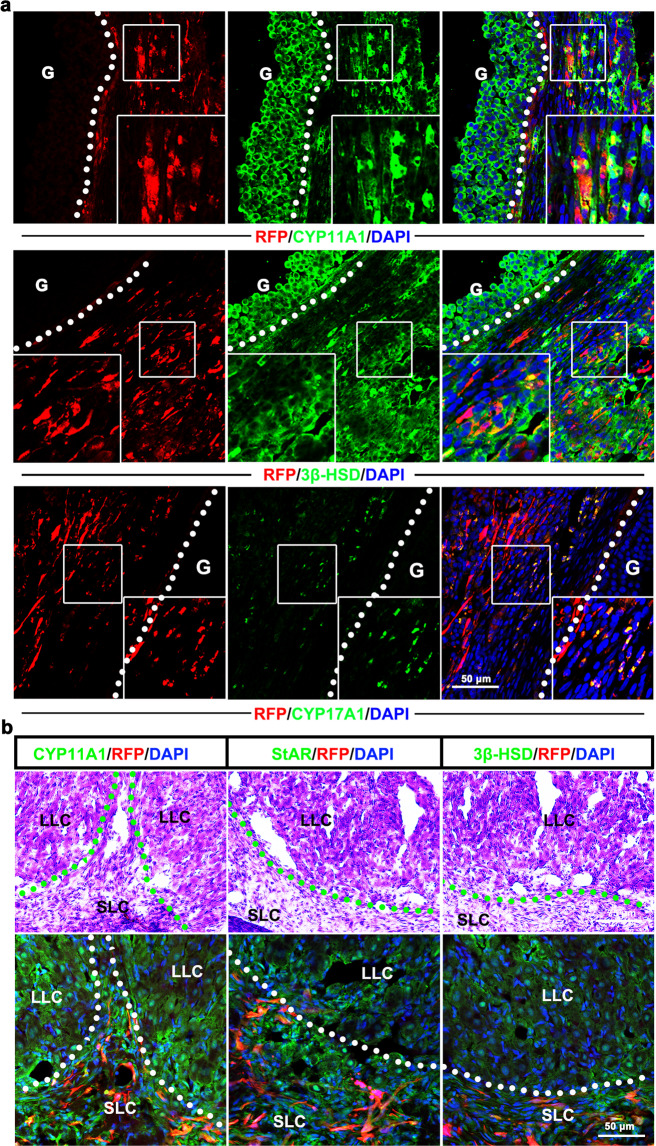
Transplanted TSCs regenerate theca cells in the follicles of monkeys with POI. **a** CYP11A1, 3β-HSD, and CYP17A1 coexpressed with RFP-positive cells in the theca membrane of the ovarian follicle, G granulose cells. The staining for CYP11A1 and 3β-HSD was observed not only in the theca cell layer but also in granulosa cells in large antral follicles. **b** CYP11A1, StAR, and 3β-HSD co-expressed with RFP-positive cells in the corpus luteum, LLC large lutein cells, SLC small lutein cells.

To investigate the fate of the transplanted TSCs in the ovary, we transduced the cells with a lentiviral vector expressing red fluorescent protein (RFP) driven by the CAG promoter. Fluorescence microscopy detection showed that RFP-positive cells surrounding follicles comprise more than one layer and are arranged parallel to the basement membrane in the TSCs group. These cells were also localized within the theca lutein of the corpus luteum akin to natural thecal cells in vivo (Supplementary Fig. S6a). When performed immunofluorescent staining, these RFP-positive cells specifically expressed the markers of mature TCs, such as CYP11A1 (13.33 ± 0.88%), 3β-HSD (11.00 ± 0.58%), and CYP17A1 (10.67%) (Fig. 5a and Supplementary Fig. S6b). Additionally, 10.33 ± 1.20% of Ki67^+^RFP^+^ cells were found in the ovarian sections, indicating that transplanted TSCs proliferate in vivo (Supplementary Fig. S6c, d). After ovulation, the transplanted TSCs (RFP positive cells) were found expressed CYP11A1, 3β-HSD, and StAR in the small luteal cells region to contributes to the formation and development of corpus luteum (Fig. 5b). Further, we could observe that the transplanted TSCs retained in the ovarian interstitium for about 3 months (Supplementary Fig. S6e). Whether the transplanted cells can normally contact with endogenous cells in vivo is a critical point for the long-term survival of transplanted cells^38^. We found that RFP-positive TSCs can connect with endogenous TCs through CONNEXIN 43 (Supplementary Fig. S6f). However, unlike TSCs, the RFP-positive ADSCs were randomly localized in the ovary (Supplementary Fig. S7a). Taken together, these results indicated that transplanted TSCs could differentiate, proliferate, and engraft into the host ovary of POI monkeys. These features increase the probability of ovarian follicle development and are conducive to the development of oocytes.

### Autologous transplantation of TSCs advances oocyte development in POI monkeys

**Fig. 6:**
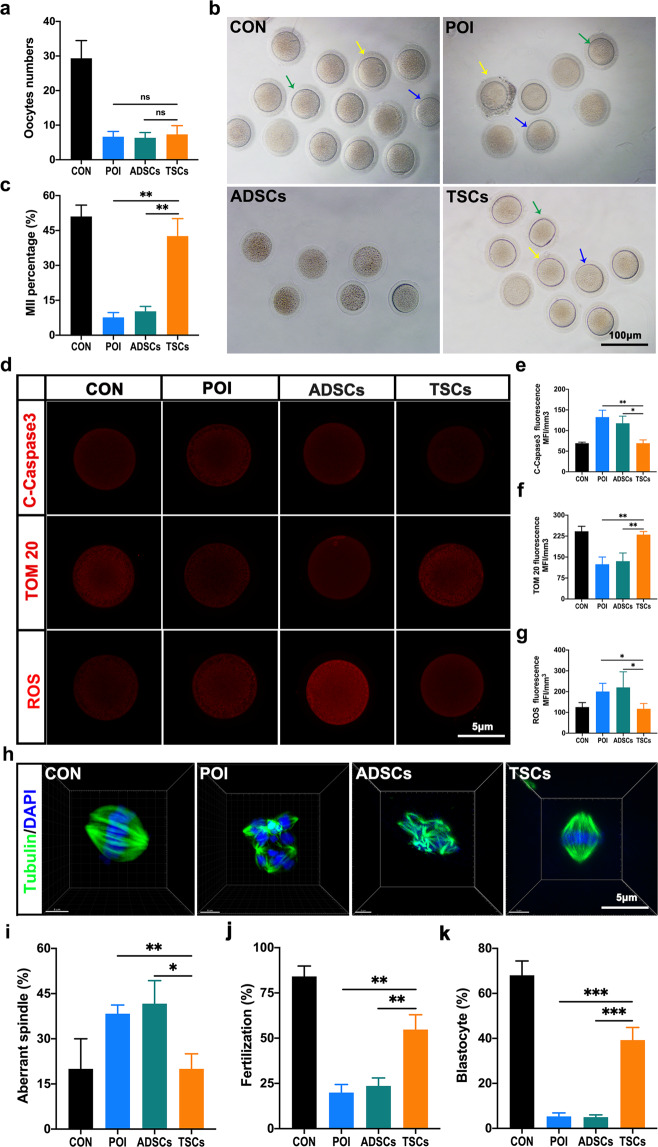
TSCs transplantation improves oocyte development and quality. **a** Statistics on the number of oocytes obtained for superovulation after TSCs transplantation. **b** Phase-contrast micrographs of oocytes in different groups. The green arrow represents MII, the blue arrow represents MI, and the yellow arrow represents GV. The number of oocytes obtained after COS. **c** Statistics on the maturation percentage of oocytes obtained for superovulation after TSCs transplantation. **d** The quality of eggs was observed by immunofluorescence staining with an anti-cleaved caspase3, TOM-20, and ROS in the oocytes. **e** Statistics analysis of cleaved caspase3 fluorescence indicating oocytes apoptosis, the density of fluorescence represents the level of apoptosis. **f** The richness of mitochondria in oocytes was observed by immunofluorescence staining with an anti-TOM20, and the density of fluorescence represents the number of mitochondria. **g** Statistics analysis of the oxygenation of the oocytes were observed by immunofluorescence staining with an anti-ROS probe, and the density of fluorescence represents the level of ROS. **h** Chromosomal behavior (anti-β-tubulin and DAPI). **i** Statistics analysis of the chromosomal behavior in the oocytes. **j** The percentage of the oocyte fertilization after ICSI in vitro. **k** The percentage of the blastocyst of the embryo after ICSI in vitro. The CON group represents the normal wild type monkeys (*n* = 3), the POI group represents the monkeys with premature ovarian failure (*n* = 3), the ADSCs group represents the monkeys with POI that received adipose-derived mesenchymal stem cells transplantation (ADSCs = POI + ADSCs, *n* = 3); the TSCs group represents the monkeys with POI that received thecal stem cells transplantation (TSCs = POI + TSCs, *n* = 3). Data were expressed as the means ± SEM and significance were determined by one-way ANOVA. *P < 0.05, **P < 0.01, ns not significant.

We then tested whether the fertilization capacity of oocytes is improved after TSCs transplantation. After superovulation, we found that the dominant follicles and the transparency of the ovary in the TSCs group were not obvious, and the total number of oocytes did not improve significantly in the TSCs group (Fig. 6a; Supplementary Fig. S8a). However, the oocyte maturation rate (43.33 ± 4.63%) was significantly increased in this group compared with the ADSCs group (10.67 ± 0.88%) and close to the control group (51.00 ± 3.22%) (Fig. 6b, c). Oocyte quality affects early embryonic survival and blastocyst formation and is evaluated based on mitochondria number, oxidative stress, and spindle structure^39^. Using immunohistochemistry, we demonstrated that the cleaved caspase3 expression in the oocytes in the TSCs group (69.24 ± 4.67 MFI/mm^3^) was significantly reduced compared with that in the ADSCs group (117.70 ± 9.96 MFI/mm^3^). The number of mitochondria in the TSCs group (230.00 ± 6.46 MFI/mm^3^) was distinctly increased compared with the ADSCs group (135.30 ± 16.88 MFI/mm^3^). Moreover, the ROS level associated with oxidative damage was significantly reduced in the TSCs group (117.30 ± 16.79 MFI/mm^3^) compared with the ADSCs group (220.20 ± 43.44 MFI/mm^3^) (Fig. 6d–g). More importantly, we found that the proportion of oocytes with disorganized spindle apparatuses were notably reduced in the TSCs group (20.00 ± 2.89%) compared with the ADSCs (41.68 ± 4.41%) group (Fig. 6h, i).

Furthermore, when we performed intracytoplasmic sperm injection (ICSI), we found the fertilization rate (54.33 ± 3.48%) and blastocyst rate (41.01 ± 2.08%) of the TSCs group were increased compared with those of the ADSCs group (fertilization rate: 23.68 ± 2.33%; blastocyst rate: 4.00 ± 0.58%) (Fig. 6j, k and Supplementary Fig. S8b). Taken together, TSCs transplantation strikingly improve oocyte maturation and quality and may benefit for fertility.

## Discussion

TSCs have been identified and isolated in several species, including humans, but preclinical studies of TSCs transplantation to relieve the relevant symptoms of POI have not been reported. Here, we first establish a method for the isolation of TSCs from NHPs and demonstrate that cynomolgus monkey TSCs have the capacity to partially restore hormone production, and improve the ovary function in vivo following transplantation into POI animal models.

Previous studies have demonstrated that TSCs could be isolated from a completely removed ovary, and most of the ovaries were obtained from neonatal or healthy individuals, which is infeasible for autologous TSCs transplantation^23,40^. TSCs are thought to be located in the cortical layer of the ovary^21,41^. Therefore, a major concern regarding is whether the TSCs can be produced on a clinical scale using small amounts of the cortical layer. Additionally, although the enzymatic process is currently the most widely used to isolate stromal cells, enzymes, such as collagenase, may be associated with concerns regarding their clinical safety. Chaput et al. also found that mechanical isolation of the stromal vascular fraction yielded three-fold more colony-forming units than enzymatic digestion and centrifugation techniques^42^. In this study, we successfully established an optimization scheme to obtain monkey TSCs from a small piece of the ovarian cortical layer by using a mechanical and enzyme-free isolation method. Cells can be cultured to a large scale (more than 1.0 × 10^11^ cells at P10) in a system similar to SLCs. They also can maintain their stemness characteristics and express SLCs-specific markers such as NESTIN, CD271, and PDGFRα, which reveals that they might be the putative specific cell markers for TSCs. Furthermore, when TSCs were cocultured with GCs in the induction system, they could differentiate into mature TCs producing DHEA and A2. Therefore, we determined that the TSCs acquisition method and culture system were feasible and effective.

NHPs share many similarities with humans based on ovarian morphological characteristics, menstrual cycle, and patterns of sex hormone secretion are the same as human, which makes them an ideal animal model for laboratory and preclinical studies in POI^25,43,44^. Currently, most POI models are chemically induced to mimic the POI caused by chemotherapy treatment^45^, but it seems that appropriate doses and action times are very important for the success of POI model establishment. In addition, chemical-induced ovarian damage can be spontaneously restored in an animal model, depending on the timing of cessation of chemical exposure^46^. Therefore, the inconsistency and variation of pathological features in this model restrict its application to mimic spontaneous POI caused by aging. Unlike many poison-induced POI models, we analyzed the repeated ovarian stimulation effect on oocyte donor cynomolgus monkeys and generated a POI monkey model similar to that reported by Nie et al. reported in rodents^29^. Hormones levels, follicle pool, and ovarian function of these monkeys were changed similar to the pathological characteristics of clinical POI patients, which is diagnosed by the reduction of E2, AMH, and AFC and increase in FSH secretion^1,29^. Thus, these POI monkey models may well simulate the pathophysiological state of the complex process of ovarian aging and be suitable for further estimating the cell therapeutic strategy.

Many studies have demonstrated that TSCs are recruited and differentiated into TCs, forming the follicular membranes surrounding the GCs to provide structural support and contribute to steroidogenesis for follicle maturation^21,47^. Lu et al. reported that human umbilical cord-derived mesenchymal stem cell transplantation can restore the ovarian function by alleviating theca-interstitial cells apoptosis in POI rats^48^. We hypothesized that TSCs were more suitable for POI therapy as tissue resident stem cells. By considering TSCs have shown the characteristics of mesenchymal stem/stromal cells, ADSCs were taken to be a control. By tracking the transplanted cells, in contrast to the random distribution of ADSCs, we detected that most of the donor TSCs-derived TCs participated in the structure of the fully growing follicle and remodeled gap junctions with the local cells. Furthermore, TSCs transplantation also significantly increased the serum DHEA levels, indicating that these cells could potentially ameliorate the hormone levels by increasing the TCs function in POI. Additionally, after ovulation, the TCs and GCs will differentiate into small and large luteal cells respectively, which had the ability to produce androgens and support pregnancy^17,49,50^. In our study, we also observed that TSCs could retain at the outer edge of the corpus luteum such as small luteal cells did and the TSCs group has a more distinct corpus luteum (data not shown). This finding indicated that TSCs transplantation may also have important implications for the improvement of fertility.

Additionally, we only found that partly RFP-positive cells co-expressed the markers of mature TCs. The results of RNA-Seq revealed certain degree of similarities between TSCs and ADSCs (correlation coefficient, *R*^2^ = 0.7288) in PluriNet genes (Supplementary Fig. S9a). However, further functional analysis of differential expressed genes showed that TSCs were more active in the pathways of organismal survival, fatty acid metabolism, and differentiation of stem cells (Supplementary Fig. S9b). Besides, we also observed that the concentration of free fatty acid (FFA) in follicle fluid was remarkably decreased in the TSCs group and the apoptosis of GCs was significantly lower in TSCs group (15.57 ± 1.497%) than that in ADSCs group (28.33 ± 1.938%) (Supplementary Fig. S9c–e). Both of these results hint that TSCs might have an impact on the GCs survival and FFA metabolism to promote follicle development directly, indicating that tissue-resident stem cells may be more suitable for their original tissue repair. Further investigation is needed to uncover the exact role of TSCs, whether TSCs are replenished exclusively by differentiating into TCs or they may promote endogenous TSCs differentiation and cell survival directly.

In summary, we first reported the comprehensive study of TSCs in the cynomolgus monkeys and increased our understanding of the important roles of TSCs in the ovary and clinical application prospects. More excitingly, we reveal that autologous TSCs transplantation can improve hormone levels, promote follicular development, and replenish oocytes in POI monkeys. Advantageously, in the long-term observation after TSCs transplantation, we did not observe immune rejection or tumors in the animals. Thus, TSCs transplantation is a potential treatment for POI infertility in the future and a nonhuman primate animal model with POI may act as an important model for evaluating cell transplantation therapy for the treatment of female infertility.

## Materials and methods

### Animals

Eight-week-old male NOD-Prkdc^em26Cd52^ Il2rg^em26Cd22^/Nju (NCG) mice (the Model Animal Research Center of GemPharmatech Co., Ltd) were maintained under controlled temperature (24°C ± 1°C) and relative humidity (50%–60%) conditions with a 12-h light/12-h dark cycle for the tumorigenicity assay. Free access to standard rodent diet and drinking water was provided. Fourteen female cynomolgus monkeys (age 8–13 years) and one male cynomolgus monkey were selected and housed at the Blooming Spring Biological Technology Development Co., LTD, which is fully accredited by the Association for Assessment and Accreditation of Laboratory Animal Care International (AAALAC). All animals were fed a commercial monkey diet twice per day plus one meal of seasonal fruits daily with free access to clean water, and under careful veterinary monitoring to evaluate and ensure their health status daily. We found that the number of retrieved oocytes and quality decreased significantly after several rounds of controlled ovarian stimulation (COS) in some of the oocyte donor monkeys. The cynomolgus monkey models of POI were screened by statistical observation of the menstrual cycle, serum hormone assay, and oocyte monitoring. All procedures complied with the guidelines of Sun Yat-sen University and were approved by the Ethics Committee of Sun Yat-sen University. Animal grouping and specific information are listed in Supplementary Table S2.

### Cell culture

To prepare TSCs, a small piece of ovarian cortex (~1% of the ovary volume) was cut by laparoscopy, transferred to the prepared medium, and washed thrice with phosphate-buffered saline (PBS, Thermo Fisher). The tissue was dissected into small pieces with scissors, resuspended in TSCs medium and then transferred into a 12-well plate with fresh medium in a 37 °C and 5% CO_2_ incubator. After 24 h, the cell suspension was collected and transferred into a new culture well, and then the medium was changed every 2 days until the cells reached 90% confluence. The cells were passaged and expanded according to the protocol for the establishment of TSCs with slight modifications by using SLC medium^33,34^. The TSCs medium consisted of DMEM/F12 (1:1; Gibco) supplemented with 1 nM dexamethasone (Sigma), 1 ng/mL LIF (Millipore), 5 μg/L insulin-transferrin-sodium selenite (ITS, Sigma), 5% chicken embryo extract (US Biologicals), 0.1 mM-mercaptoethanol (Invitrogen), 1% nonessential amino acids (HyClone), 1% N2 and 2% B27 supplements (Invitrogen), 20 ng/mL basic fibroblast growth factor (Pepro Tech), 20 ng/mL epidermal growth factor (Pepro Tech), 20 ng/mL platelet-derived growth factor-BB (Pepro Tech), 2% FBS (HyClone), and 20 ng/mL oncostatin M (Pepro Tech). The cultures were maintained at 37 °C in a humidified 5% CO_2_ water-jacketed incubator. The medium was changed every 2 days.

ADSCs were obtained from the inguinal subcutaneous adipose tissue of female monkeys. In brief, the epidermal hair was removed from the skin, disinfected with iodine thrice with iodine disinfection and alcohol deiodination, and then rinsed with normal saline thrice. Adipose tissue was harvested and diced using scissors followed by digestion with 0.1% type I and IV collagenase (Sigma) in serum-free medium at 37 °C for 30 min. The DMEM-low glucose (Gibco) with 10% fetal bovine serum (FBS) was used to stop the tissue digestion and debris was removed by filtering. The samples were then centrifuged at 1200 rpm for 3 min and rinsed twice with PBS. The pellets were cultured in DMEM-low glucose supplemented with 10% FBS, 50 U/mL penicillin, and 50 μg/mL streptomycin in a humidified incubator at 37 °C with 5% carbon dioxide. The medium was changed every 2 days.

The GCs were prepared from follicles in the ovaries of superovulated female cynomolgus monkeys^27^. TCs and oocytes were removed as previously reported^21^. The GCs were washed thrice with the DMEM/F12, then resuspended in cultivation medium and seeded. GCs expansion medium consisted of DMEM/F12 with 10% FBS, 10 mg/mL ascorbic acid (Sigma-Aldrich, USA), 0.05 μM dexamethasone (Sigma-Aldrich, USA), 200 mM l-glutamine, 10 mg/mL gentamicin, 10,000 units/mL penicillin, and 10,000 μg/mL streptomycin. The cultivation medium was changed every 2 days. Cells were cultivated at 37 °C with 5% CO_2_.

### Flow cytometry analysis

For FACS analysis, the cells were cultured in vitro and amplified. TSCs or ADSCs were harvested and suspended in PBS (1 × 10^6^ cells/mL), and then all these cells were incubated in BD Cytofix/Cytoperm (BD Biosciences, USA). We stained the cells with fluorochrome-conjugated antibodies in the dark for 30 min at 4 °C. The samples were centrifuged at 1100 rpm at 4 °C for 4 min. Then, the pellet was washed with PBS and finally analyzed by BD FACSCanto II Flow cytometer (BD Biosciences, USA) or the BD LSRFortessa cell analyzer flow cytometer (BD Biosciences, USA).

### Theca cell differentiation

To investigate the differentiation of TSCs into TCs, the TSCs (1.0 × 10^5^ cells) were cultured with GCs (1.0 × 10^5^ cells) in differentiation-inducing medium for 12 days as previously reported^21^. The inducing medium contained 15% FBS, 10 ng/mL PDGF-AA (Pepro Tech), 1 ng/mL LH (Pepro Tech), 1 nM thyroid hormone (Pepro Tech), and 50 ng/mL insulin-like growth factor 1 (IGF1, Pepro Tech) in TSCs basic medium. The medium was changed every 3 days. Differentiation was subsequently confirmed by RT-PCR and immunostaining for TCs lineage-specific genes. The culture supernatant was also collected for DHEA, A2, and E2 detection by biochemical immunoassay (ARCHITECT *i*2000SR, Abbott Laboratories, USA) on specified days (days 3, 6, 9, and 12).

### Osteogenic differentiation

TSCs were cultured in osteogenesis-inducing medium containing 20% FBS, 100 μg/mL ascorbic acid (Sigma), 100 nM dexamethasone (Sigma), 10 mM β-glyc-erophosphate (Sigma), and 100 IU/mL penicillin/streptomycin (Invitrogen) in α-MEM (Invitrogen) for 4 weeks, and the inducing medium was changed every 3 days. Then, TSCs-derived osteogenic-differentiated cells were fixed and stained with Alizarin red S to detect the presence of calcium as previously described^51^.

### Adipogenic differentiation

TSCs were incubated in high-glucose DMEM containing 100 nM dexamethasone (Sigma), 10 μg/mL insulin (Sigma), 0.2 mM indomethacin (Sigma), 0.5 mM 3-isobutyl-1-methylxanthine (Sigma), 10% FBS and 100 IU/mL penicillin-streptomycin for 4 weeks and inducing medium was changed every 3 days. The TSCs-derived adipogenic-differentiated cells were confirmed by Oil red O staining as described previously^51^.

### Chondrogenic differentiation

As previously reported, chondrogenic differentiation was induced using a cell pellet culture system. First, 2 mL of TSCs suspension was maintained in a 15-mL conical tube with the induction medium consisting of DMEM (Invitrogen) with 3% FBS, 10 ng/mL tumor growth factor (TGF)-β3 (PeproTech), 1× ITS (Sigma), and 1 mM pyruvate (Sigma) for 4 weeks. The inducing medium was changed every three days. Finally, the TSCs derived chondrocytes were identified by toluidine blue (Sigma) staining, as described previously^51^.

### Lentiviral vector infection

For TSCs or ADSCs tracing, 1.0 × 10^6^ cells were treated with lentivirus (Vigene Biosciences) expressing red fluorescent protein (RFP). The lentiviral expression vector was designated pLent-CAG-mcherry-P2A-Puro. Detailed viral treatments were performed according to a previous report^33,51^.

### Cell transplantation

For cell transplantation, TSCs or ADSCs were digested and suspended at approximately 2.5 × 10^8^ cells/mL in 160 µL PBS, and then the cell transplants were performed by laparoscopy. For this purpose, we used grasping forceps to hold the ovary ligament and fix the ovary and a 21G spinal needle to inject the cells into each ovary.

### Sperm, oocyte collection

Monkey semen and oocytes were collected and washed with TL-HEPES as previously described^27^. Female cynomolgus monkeys were intramuscularly injected with rhFSH (recombinant human follitropin alfa; GONAL-F, Merck Serono) daily for 8 days followed by rhCG (recombinant human chorionic gonadotropin alfa; OVIDREL, Merck Serono) on day 9. Oocytes were collected after rhCG administration for 33–36 h. Metaphase II (MII, the first polar body is present) oocytes were collected and cultured in CMRL-1066 medium containing 0.1% Na-lactate (Sigma, L1375) and subjected to intracytoplasmic sperm injection (ICSI) or immunofluorescence for quality detectin. The zygotes were cultured in embryo culture medium-9 (HECM-9) containing 10% fetal bovine serum (FBS; HyClone Laboratories, SH30088.02) at 37 °C in 5% CO_2_.

### Sex hormone assay

Cell culture supernatants and serum were collected at the indicated time points. Blood samples were held for 90 min at room temperature, and serum was obtained after centrifugation (3000 × *g*, 15 min). Serum was stored at −80 °C until the hormone was assayed. Serum AMH, InhB3, E2, P, DHEA, and A2 were analyzed using biochemical immunoassays (Beckman, AU5800, USA). FSH, was analyzed by ELISA kits according to the manufacturer’s guidelines. The optical density (OD) value was determined at a wavelength of 450 nm by using an ELISA microtiter plate reader (Tecan, USA). The hormone concentrations were calculated using a standard curve as described in the package insert of the ELISA kits.

### Ultrasound

B-ultrasonic examination was performed before and after cell transplantation to evaluate the animals’ cardiac function, the number of AFCs, and the uterine and ovarian volumes. Macaques were sedated with Zoletil 50 (Virbac S.A) and then were examined in the supine position, using a commercially available ultrasound system (ALOKA Prosound α7). Apical four-chamber views were collected for baseline left ventricular ejection fraction (LVEF) measurement. Quantification of LVEF was performed by a qualified cardiologist according to the recommendation of the American Society of Echocardiography^52^. The volume of the uterus and the ovary were measured by simulated clinical methods as reported^53^.

### Ovarian follicle counting

Ovaries were collected and fixed in 4% paraformaldehyde for 12 h, embedded in paraffin, serially sectioned at a thickness of 5 μm, and then stained with hematoxylin and eosin. The follicles were counted with a visible nucleus every second section. Follicle classification was determined by Pederson’s standard. Specifically, primordial or primary follicles were defined as the oocytes surrounded by a single layer of flattened or cuboidal granulosa cells; secondary follicles were defined as oocytes surrounded by more than one layer of cuboidal granulosa cells with no visible antrum. A clear antral space and a cumulus granulosa cell layer were defined as mature follicles^54^. The results were reported as the number of follicles counted per ovary.

### Immunofluorescence

For immunofluorescence staining, the cells were treated as described previously^33,51^. The cells were fixed with 4% paraformaldehyde for 15 min and permeabilized with 0.3% Triton X-100 (Sigma) for 15 min and blocked with 3% bovine serum albumin (BSA, Sigma) for 20 min at room temperature. The cells were incubated overnight with the primary antibodies and blocked with 1% bovine serum albumin (BSA, Sigma) at 4 °C. Moreover, they were incubated for 45 min with the appropriate secondary antibodies after washing thrice with PBS. Nuclei were counterstained with DAPI (Thermo Fisher) for 5 min at room temperature.

For immunofluorescence staining of ovarian tissues, the tissues were fixed in 4% paraformaldehyde overnight, dehydrated with 30% sucrose and sectioned at a thickness of 10 or 50 μm after embedding with OCT (SAKURA). The sections were permeabilized for 15 min using 0.3% Triton X-100 (Sigma), incubated overnight with the primary antibodies and blocked with 3% BSA (Sigma) in PBS. Then, sections were incubated with the appropriate secondary antibodies for 45 min at room temperature after washing thrice with PBS, and 5 min with DAPI (antibodies are listed in Supplementary Table S3)^33,51^.

For immunofluorescence staining of oocytes, the eggs were fixed in 1% paraformaldehyde for 60 min and 0.5% Triton X-100 at 37 °C for 60 min. Then the oocytes were incubated in phosphate-buffered saline (PBS) with 0.5% Triton X-100 (PBT) at 4 °C for 60 min and blocked in PBT with 5% BSA (PBT-BSA) overnight at 4 °C. All primary antibody incubations were performed in PBT-BSA at 10 mg/mL overnight at 4 °C, washed thrice with PBS and then incubated with the appropriate secondary antibodies for 45 min. Nuclei were counterstained with DAPI (Thermo Fisher) for 5 min at room temperature^55^. Images were captured with an LSM800 confocal microscope (Zeiss) or LSM880 confocal microscope (Zeiss) and a Dragonfly CR-DFLY-202 2540 (Andor Technology).

### Measurement of ROS levels

Intracellular ROS levels were determined by CM‐H2DCFDA (Cat# S0033; Beyotime Biotechnology TM). CM‐H2DCFDA was prepared in DMSO prior to loading. Oocytes were incubated in PBS containing 5 mM CM‐H2DCFDA for 20 min at 37 °C in a 5% CO_2_ incubator^54^. After washing thrice, oocytes were loaded on a slide with a micro drop of medium and immediately observed under a laser scanning confocal microscope (LSM 880, Zeiss).

### RT-PCR analysis

The fraction of total RNA was extracted using a RNeasy Mini kit (QIAGEN) and reverse-transcribed using the SuperScript III First-Strand Synthesis System (Invitrogen). Quantitative PCR was performed with Power SYBR (Applied Biosystems) according to the manufacturer’s instructions. The gene expression level was normalized to that of *GAPDH* using the ∆∆Ct method. The primers used for the reactions are as follows (Supplementary Table S4).

### RNA-seq and bioinformatic analyses

Total RNA was isolated using TRI Reagent (#TR118, Molecular Research Center, Inc.) following the manufacturer’s instructions. RNA samples with an RNA Integrity Number > 8 were selected to perform library-preparing and paired-end sequencing with the standard protocol by CapitalBio Technology (China). Illumina sequencing output generated bcl files were de-multiplexed by bcl2fastq2. De-multiplexed read sequences were then aligned to the Crab-eating macaque Genome macFas5, assembly and differential expression was estimated using DESeq2^56^. The functional enrichment analysis of differential expressed genes (fold changes > 1.5) between ADSCs and TSCs was performed through Ingenuity Pathway Analysis (IPA). The gene lists enriched in the selected cell properties were acquired from Human Protein Atlas (HPA) database and references^57–59^.

### Tumorigenesis assay

The P5 and P10 of monkey TSCs at different cell numbers (1.0 × 10^4^, 1.0 × 10^5^, 1.0 × 10^6^, and 1.0 × 10^7^ cells), ADSCs and MLTC-1 Leydig tumor cells at P5 were suspended in 200 µL of a 1:1 mixture of growth-factors reduced Matrigel: PBS and injected subcutaneously into NCG mice. For all cells injected, the tissues were harvested up to 8 months unless a tumor was formed earlier. The tissues were fixed overnight in cold 4% paraformaldehyde, rinsed twice with PBS, soaked in 30% sucrose overnight, embedded in OCT and sectioned at 10 or 50 µm.

### Karyotype analysis

G-band chromosomal analysis was performed at DAAN Gene Co., Ltd.

### Hematological test

To evaluate the safety of TSCs acquisition and stem cell transplantation therapy, the hematological parameters, including white blood cells, neutrophils, lymphocytes, monocytes, eosinophils, basophils, red blood cells, hemoglobin, and platelets were detected at time points which described in the table (Supplementary Table S1). All laboratory measurements were performed by standard autoanalyzer methods as reported (Sysmex, XN9000, Japan)^33^.

### Statistical analysis

All results represent data from at least three independent experiments and values were expressed as the mean ± SEM. Statistical analyses were performed using GraphPad Prism software (v8.0). All statistical comparisons were made using a two-tailed Student’s *t*-test or one-way ANOVA, and the threshold for significance was set at *P* < 0.05.

## Supplementary information

Supplementary Information

Supplementary Excel S1
