# Supplementary material for: Autologous transplantation of thecal stem cells restores ovarian function in nonhuman primates
Source: Cell Discov. 2021 Aug 31;7:75. doi: 10.1038/s41421-021-00291-0 (PMC8405815; doi:10.1038/s41421-021-00291-0)
Supplement: Supplementary file 1 — Supplementary Information [file 41421_2021_291_MOESM1_ESM.pdf]

Supplementary Materials

Autologous transplantation of thecal stem cells restores ovarian function in nonhuman primates

Hong Chen[1†], Kai Xia[1†], Weijun Huang[1, 2†], Huijian Li[1], Chao Wang[1], Yuanchen Ma[1], Jianhui Chen[3], Peng Luo[4], Shuwei Zheng[1], Jiancheng Wang[5], Yi Wang[1], Lin Dong[1], Zhipeng Tan[1], Xingqiang Lai[6], Frank Fuxiang Mao[7], Weiqiang Li[1], Xiaoyan Liang[3], Tao Wang[1], Andy Peng Xiang[1, 8], Qiong Ke[1, 2*]

Correspondence to: keqiong3@mail.sysu.edu.cn (Q.K.).

This file includes:

Supplementary Fig. S1 to Fig. S9

Supplementary Table S1 to Table S4

Supplementary Excel S1

a

Assessment content	Pre	Post
**Body weight (kg)**	4.203±0.61	4.213±0.64
**LV ejection fraction (%)**	75.63±3.89	75.30±2.68
**Uterus volume (cm^3)**	12.60±0.81	12.80±1.44
**Ovrian volume (mm^3)**	97.67±6.15	96.58±7.23
**FSH (mIU/ml)**	8.90±0.26	9.07±0.12
**Estradiol (pg/ml)**	143.73±24.25	152.67±20.94
**AMH (ng/ml)**	4.73±0.65	4.96±1.05

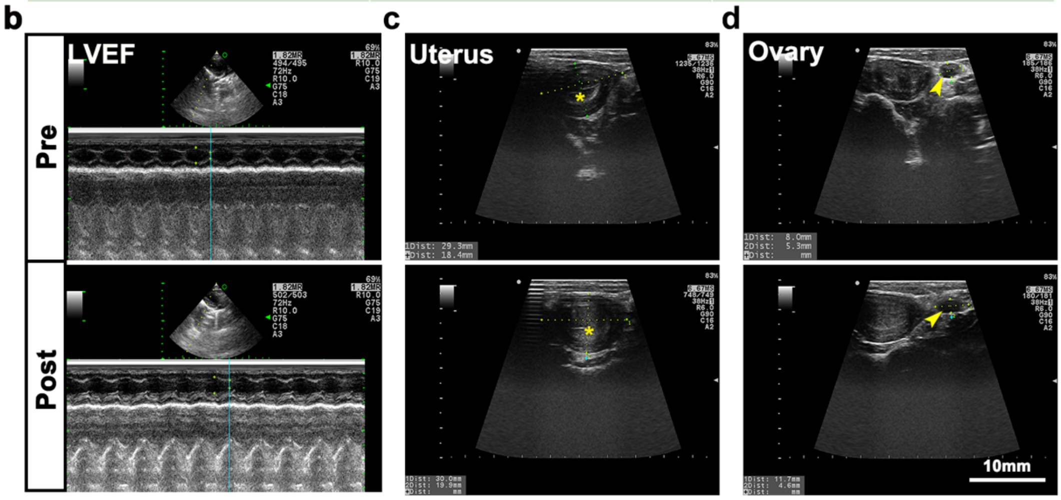

Supplementary Figure S1. The safety assessment of TSCs in vitro obtained.

(a) The safety of sampling was evaluated by physiological assessment (n = 3). Physiological indexes such as (b) LV enjection fraction (%), (c) uterus volume and (d) ovary volume were detected by ultrasound before and after sampling. Data are expressed as the means ± s.e.m.

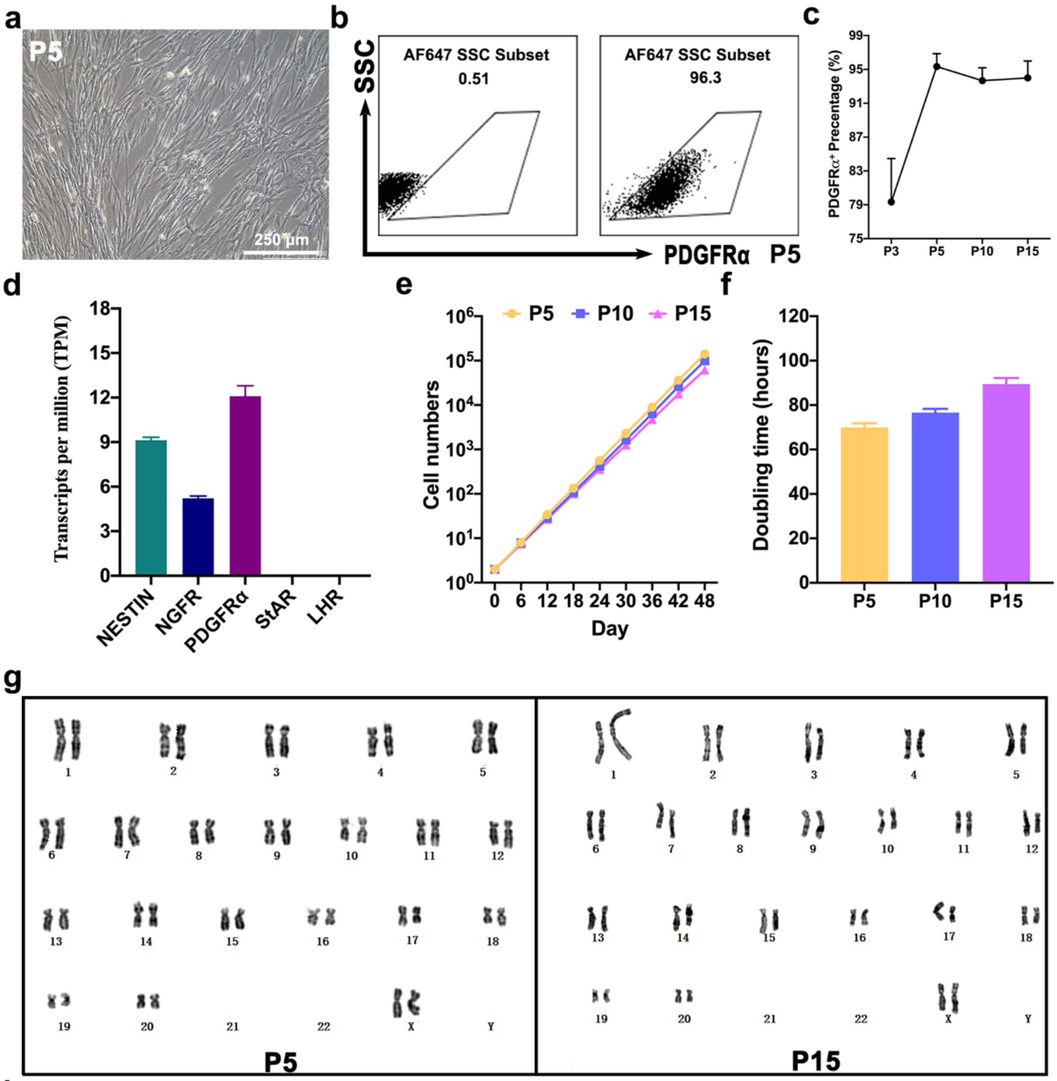

h

Cell	1× 10^4	1× 10^5	1× 10^6	1× 10^7
MA-10	3/3	3/3	/	/
ADSCs P5	0/3	0/3	0/3	0/3
TSCs P5	0/3	0/3	0/3	0/3
TSCs P10	0/3	0/3	0/3	0/3

Supplementary Figure S2. TSCs in vitro culture and safety assessment.

(a) Phase-contrast micrographs of TSCs at P5. (b) Flow cytometry was used to isolate PDGFRα$^+$ TSCs at P5. Left: isotype controls. Right: stained samples. SSC: side-scattered light. (c) The percentage of PDGFRα$^+$ cells were determined at different passages (P3, P5, P10, and P15) (biological replicates, n = 3). (d) Expression of stemness and differentiated marker gene in the TSCs at P5. (e) The proliferation rates of the isolated TSCs at different passages (P5, P10, and P15) were similar (n = 3). (f)

The average population-doubling times of cells at different passages (P5, P10, and P15) (n = 3). (g) Karyotypic stability of expanded TSCs at passage 5 (P5) and passage 15 (P15) was assessed. (h) Expanded TSCs and mouse MA-10 cells (Leydig tumor) were transplanted subcutaneously into immunodeficient NCG mice. Tumor formation was scored upon detection or (when no tumor had been detected) at 3 months, and the number of mice harboring tumors vs the total number of mice was scored. Data are expressed as the means ± s.e.m.

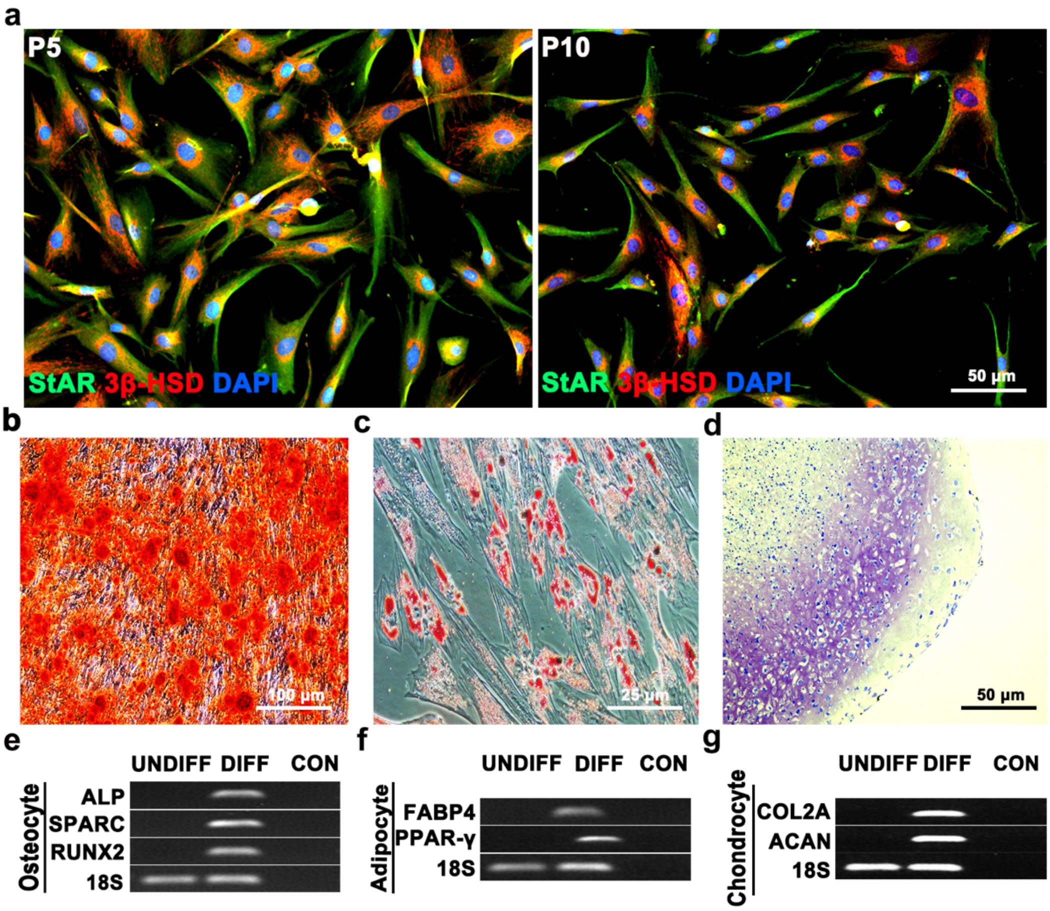

Supplementary Figure S3. TSCs differentiated into TCs in vitro.

(a) Expanded TSCs at different passages (P5, P10) were differentiated for 12 days. The cells were stained for StAR and 3β-HSD. (b) The micrographs of histological staining showing osteocytes (Alizarin Red). (c) The micrographs of histological staining showing adipocytes (Oil Red O). (d) The micrographs of histological staining showing chondrocytes (toluidine Blue). (e) RT-PCR analysis of osteogenic (*ALP, SPARC and RUNX2*) (f) adipogenic (*FABP4 and PPARγ*) (g) chondrogenic (*COL2A1 and ACAN*) cells. Markers in undifferentiated TSCs (UNDIFF) and differentiated TSCs (DIFF).

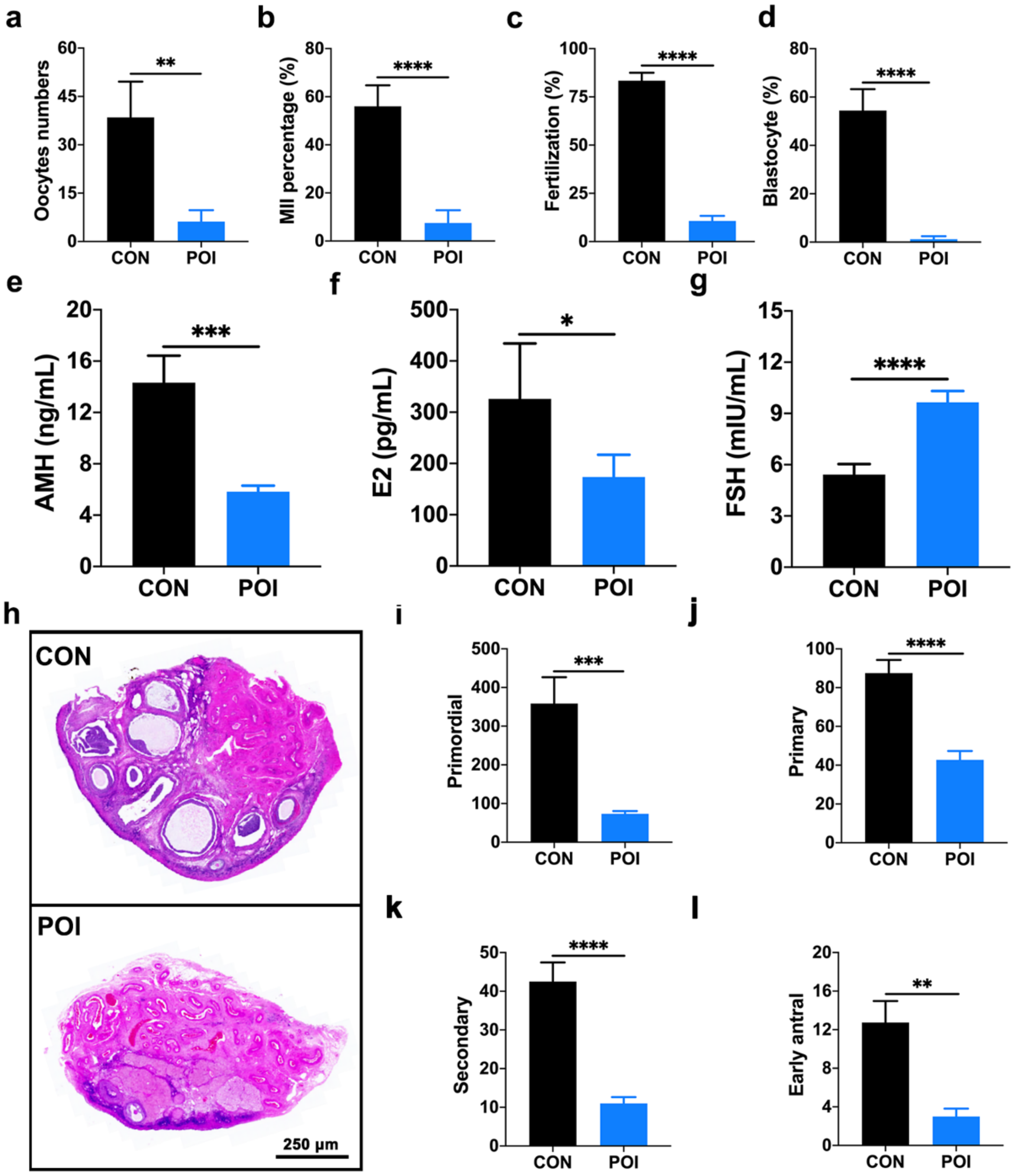

Supplementary Figure S4. The pathological manifestations of the POI cynomolgus monkey model.

(a) The statistics of oocyte number between wild type monkeys (CON) and primary ovarian insufficiency (POI) monkeys. (b) The statistics of MII percentage between CON and POI. (c) The statistics of fertilization between CON and POI. (d) The statistics of blastocyst percentage between CON and POI. Serum hormone levels such as (e) AMH, (f) E2 and (g) FSH were showed. (h) Histological analysis of ovaries between the CON and POI monkeys. (i) Primordial follicle (j) Primary follicle (k) Secondary

follicle (l) Early antral follicle (*n* = 4). CON indicates the wile type monkeys; POI indicates the primary ovarian insufficiency monkeys. Data are expressed as the means ± s.e.m and were assessed using *t*-test; * P < 0.05, ** P < 0.01, *** P < 0.001, **** P < 0.0001, ns = not significant.

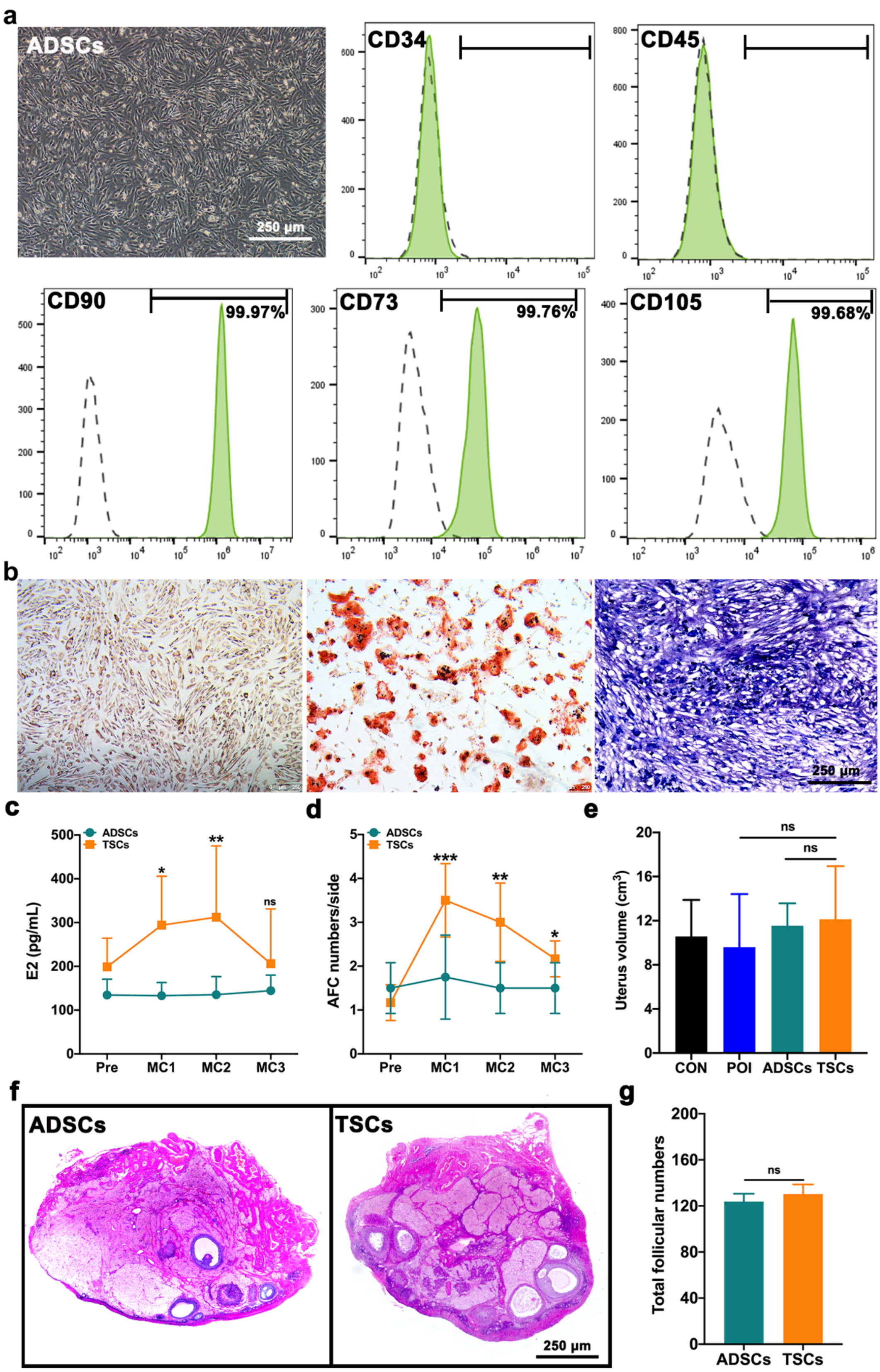

58

59 **Supplementary Figure S5. The characteristics of ADSCs and the effect of TSCs**

60 **transplantation on the ovaries of the POI monkeys.**

(a) Phase-contrast micrographs of ADSCs and the characterization by flow cytometry. More than 90% of the isolated cells were negative for CD34, CD45 and positive for CD90, CD73 and CD105. Scale bars: 100 μm. (b) Adipogenic, Osteogenic and chondrogenic differentiation of ADSCs were showed. (c) TSCs transplantation treatment can continuously improve the level of E2 for more than two months, MC = Menstruation cycle (*n* = 4). (d) TSCs transplantation treatment can continuously promote the early antral follicle development for more than two months (*n* = 4). (e) TSCs transplantation treatment had no significant effect on the uterus. (f) Histological analysis of ovaries after cell treatment of ADSCs and TSCs. (g) The number of follicles at all stages was counted, including primary, primary, secondary, and AFC (*n* = 3). The ADSCs group represents the monkeys with POI that received adipose-derived mesenchymal stem cells transplantation (ADSCs=POI+ADSCs, *n*= 4); the TSCs group represents the monkeys with POI that received thecal stem cells transplantation (TSCs=POI+TSCs, *n* = 4). Data are expressed as the means ± s.e.m and were assessed using *t*-test and one-way ANOVA. *P < 0.05. ** P < 0.01, *** P < 0.001, ns = not significant.

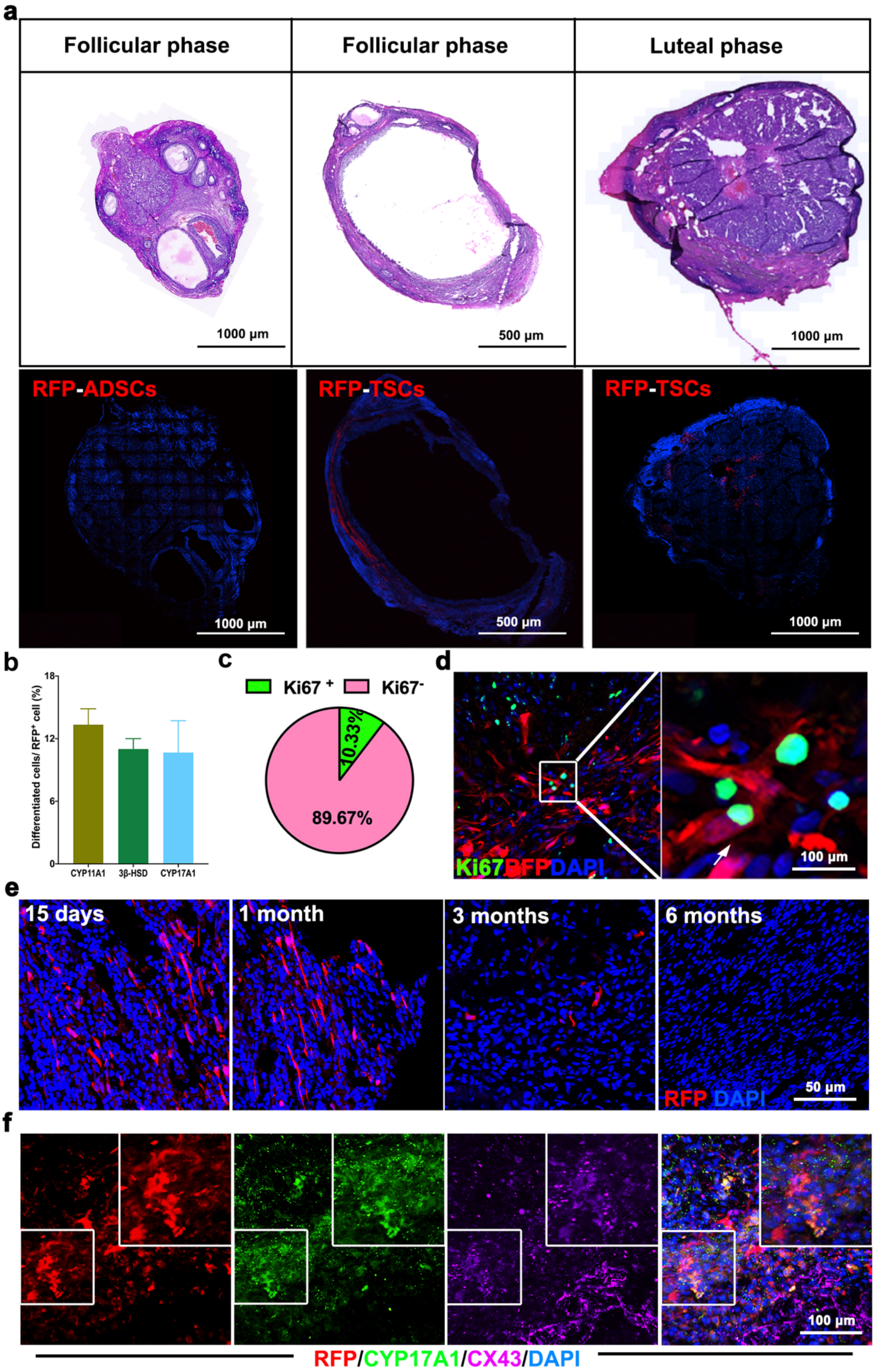

77

78 **Supplementary Figure S6. The development of TSCs transplantation in vivo.**

(a) The transplanted TSCs were involved in follicular and luteal development and ADSCs randomly distributed in the ovaries. (b) Quantitative analysis of CYP11A1, 3β-HSD and CYP17A1 positive cells from RFP$^+$ cells in the theca layers of the ovarian follicle. (c) Quantitative analysis of Ki67$^+$/ Ki67$^-$ from RFP$^+$ cells. (d) The proliferation of transplanted TSCs were demonstrated by staining for Ki67 (positive cells indicated by white arrow). (e) Cells survived in vivo after TSCs transplantation. (f) Expression of the gap junction protein connexin 43 (Cx43) in the transplanted TSCs (RFPs) and host TCs using immunostaining (indicated by pink). Data are expressed as the means ± s.e.m.

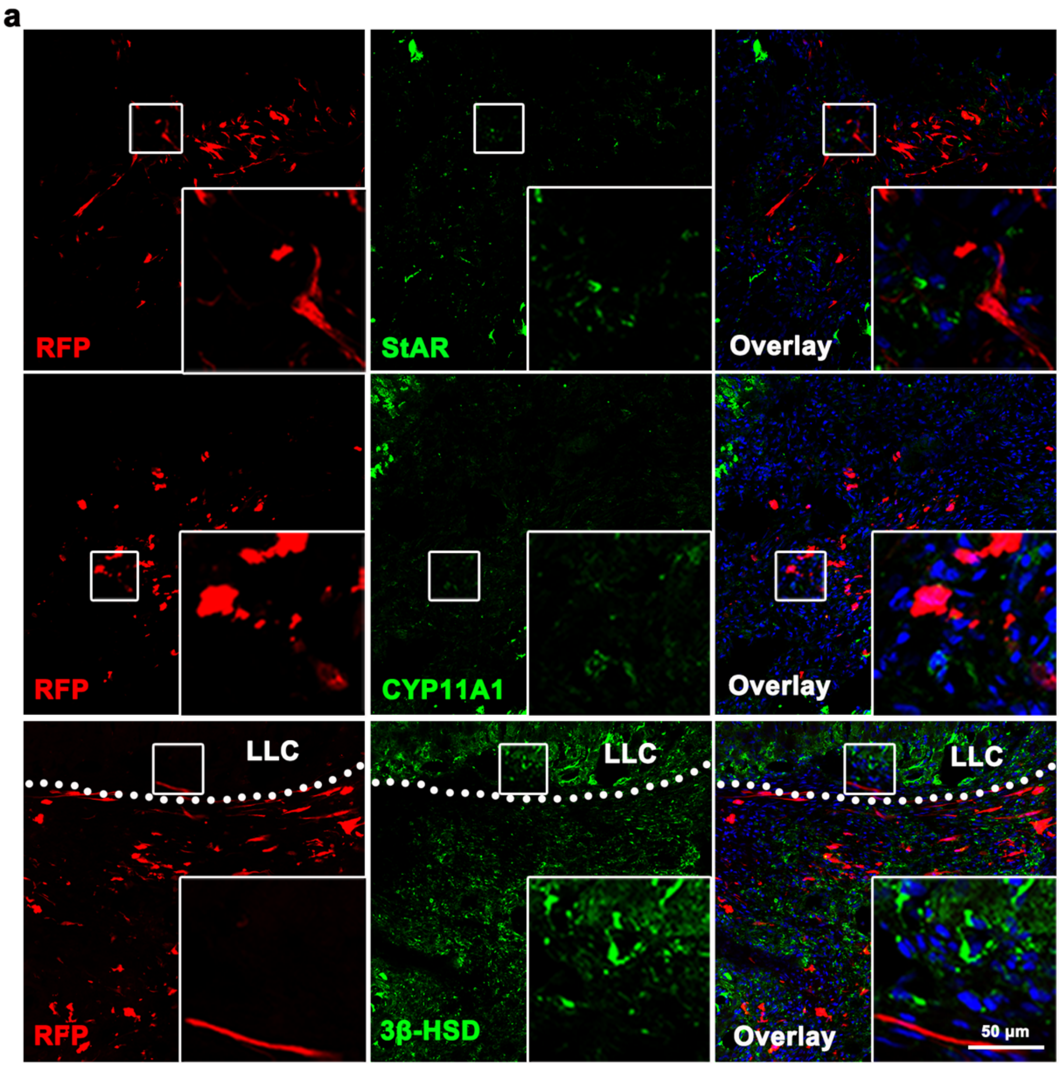

Supplementary Figure S7. The development of ADSCs transplantation in vivo.

(a) The ADSCs after transplantation are randomly distributed in the ovaries of POI monkeys and cannot express of StAR, CYP11A1, or 3β-HSD, LLC = large luteal cells.

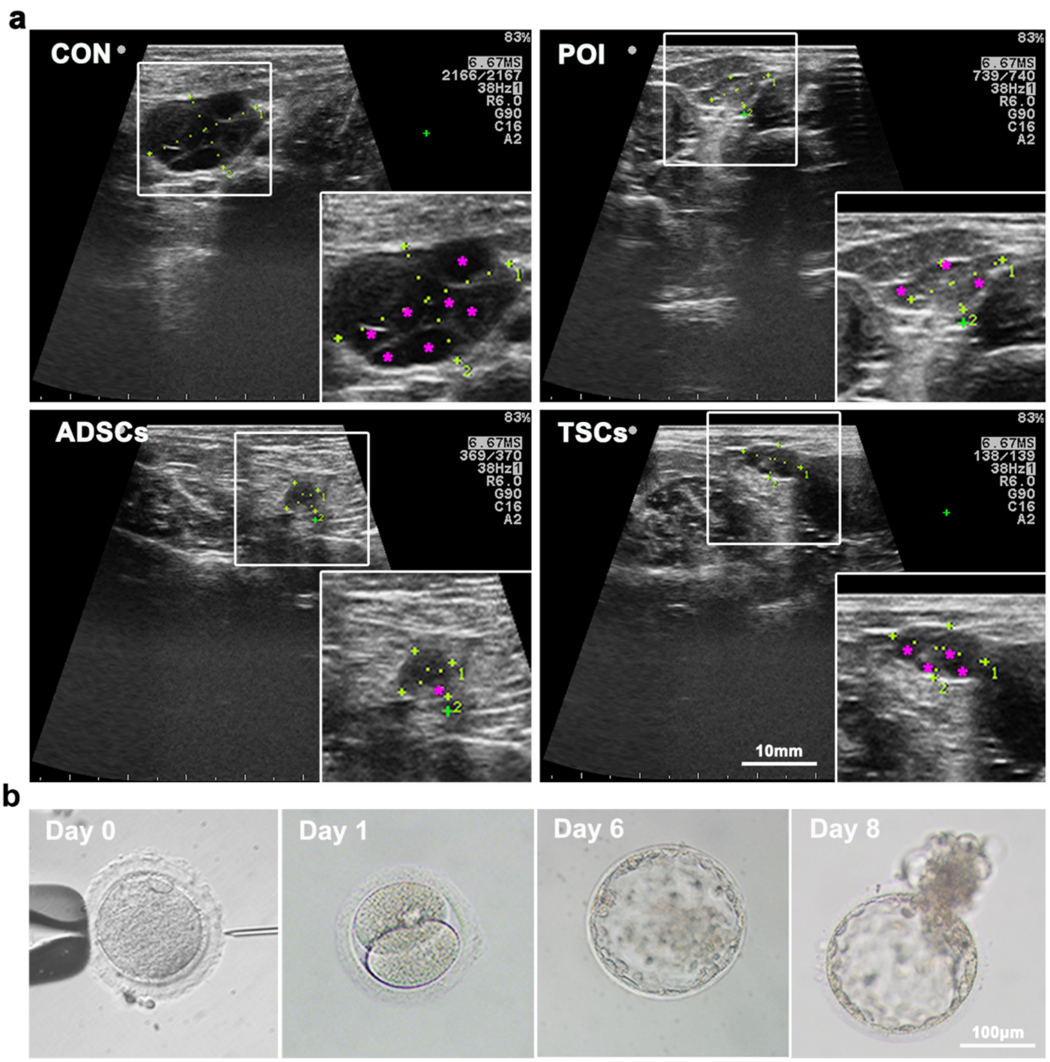

Supplementary Figure S8. The effect of TSCs transplantation on superovulation and oocyte quality.

(a) The development of ovaries after TSCs transplantation treatment had no significant stress on the exogenous hormones. (b) Oocyte fertilization and development in vitro. The CON group represents the wild type monkeys (n = 3), the POI group represents the monkeys with premature ovarian failure (n = 3), the ADSCs group represents the monkeys with POI that received adipose-derived mesenchymal stem cells transplantation (ADSCs=POI+ADSCs, n = 3); the TSCs group represents the monkeys with POI that received thecal stem cells transplantation (TSCs=POI+TSCs, n = 3).

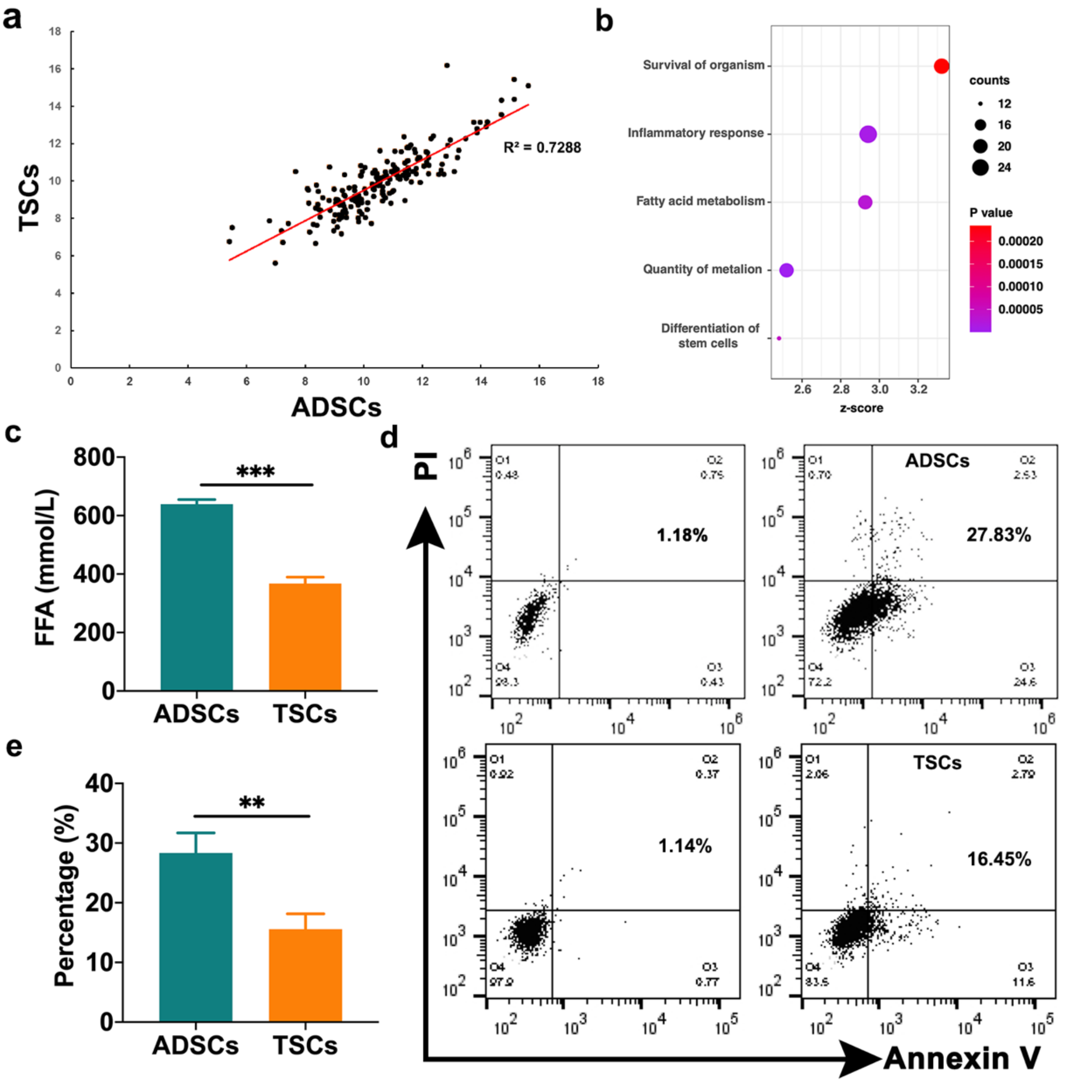

Supplementary Figure S9. TSCs transplantation promoted FFA metabolism and improved GCs apoptosis.

(a) The stemness correlation between the TSCs and ADSCs. (b) KEGG pathway analysis of the TSCs and ADSCs. (c) TSCs transplantation improves the metabolism of free fatty acid (FFA). (d) Cell apoptosis was detected by flow cytometry and statistics. (e) The percentages of cell apoptosis were detected by flow cytometry. Data are expressed as the means ± s.e.m and were assessed using *t*-test. *P < 0.05. ** P < 0.01, *** P < 0.001, ns = not significant.

Supplementary Table S1. Safety evaluation of TSCs acquisition.

	**0W**	**2W**	**4W**	**Unit**
**WBC**	9.96±0.75	10.04±0.83	9.97±0.64	$\times 10^9$/L
**Neutrophil**	4.01±0.64	3.73±0.49	4.42±0.56	$\times 10^9$/L
**Lymphocyte**	5.10±0.60	5.42±0.74	4.49±0.53	$\times 10^9$/L
**Monocyte**	0.67±0.24	0.70±0.26	0.90±0.25	$\times 10^9$/L
**Eosinophils**	0.17±0.11	0.19±0.15	0.14±0.13	$\times 10^9$/L
**Basophils**	0.01±0.05	0.01±0.03	0.01±0.05	$\times 10^9$/L
**Neutrophilv%**	38.20±1.69	38.34±1.58	44.78±1.63	%
**Lymphovyte %**	52.86±1.66	52.36±1.70	44.82±1.54	%
**Monocyte %**	6.80±0.63	7.18±0.88	8.80±0.56	%
**Eosinophils%**	2.04±0.52	2.06±0.53	1.48±0.42	%
**Basophils%**	0.10±0.13	0.06±0.09	0.12±0.12	%
**IMG**	0.06±0.09	0.04±0.06	0.06±0.05	$\times 10^9$/L
**IMG%**	0.50±0.27	0.42±0.18	0.62±0.16	%
**RBC**	5.09±0.20	5.35±0.14	5.07±0.27	$\times 10^{12}$/L
**Hemoglobin**	120.80±1.35	125.80±0.93	120.60±1.5	g/L
**Hematokrit**	39.82±0.76	42.34±0.54	39.86±0.85	%
**MCV**	78.14±0.78	79.2±0.66	78.56±0.92	FL
**MCH**	23.70±0.44	23.54±0.39	23.78±0.48	Pg
**MCHC**	303.40±1.06	297.40±0.87	302.60±0.96	g/L
**RDW-CV**	12.78±0.32	12.42±0.27	13.36±0.32	%
**RDW-SD**	35.92±0.53	34.96±0.32	37.36±0.82	FL
**Platelet**	427.60±6.66	410±5.65	439.20±4.99	$\times 10^9$/L
**MPV**	12.44±0.44	12.20±0.41	12.28±0.35	FL
**PDW**	19.02±1.25	15.94±0.66	14.98±0.49	%
**PCT**	0.52±0.22	0.49±0.19	0.60±0.15	%
**P-LCR**	46.14±1.31	42.86±1.15	44.40±1.03	%

WBC: white blood cell; **IMG**: immature neutrophils; **RBC**: red blood cell; **MCV**: Mean corpuscular volume; **MCH**: Mean corpuscular hemoglobin; **MCHC**: Mean corpuscular hemoglobin concentration; **RDW**: red blood cell distribution width; **MPV**: mean platelet volume; **PDW**: platelet distribution width; **PCT**: platelet hematocrit; **P-LCR**: large platelet cell ratio. Data are expressed as the means ± s.e.m. (*n*=5).

Supplementary Table S2. Summary of all experimental animals, treatments.

Animal Number	Gender	Age (year)	Body weight (kg)	Transplanted cell type	Transplanted cell volume (μl/side)	Number of viable cells transplanted (×10^8/sum)
M1	♀	8	4.24	ADSCs	80	2.5
M2	♀	9	5.66	ADSCs	80	2.5
M3	♀	9	3.64	ADSCs	80	2.5
M4	♀	10	3.47	ADSCs	80	2.5
M5	♀	10	3.68	ADSCs	80	2.5
M6	♀	10	5.42	ADSCs	80	2.5
M7	♀	11	3.62	TSCs	80	2.5
M8	♀	11	4.44	TSCs	80	2.5
M9	♀	11	4.43	TSCs	80	2.5
M10	♀	11	3.27	TSCs	80	2.5
M11	♀	12	4.74	TSCs	80	2.5
M12	♀	12	4.88	TSCs	80	2.5
M13	♀	12	3.51	(Sampling)	/	/
M14	♀	12	3.24	(Sampling)	/	/
M15	♂	15	6.80	(Sperm donor)	/	/

ADSCs: represents the monkeys with POI receives adipose-derived mesenchymal stem cells transplantation; TSCs: represents the monkeys with POI receives thecal stem cells transplantation; Sampling: represents the POI model monkeys ovarian acquisition.

Supplementary Table S3. Primary and secondary antibodies used for immunostaining or flow cytometry analysis.

Antibodies	Dilution	Distributor (Cat.NO)
Mouse anti-3β-HSD	1:100	Santa Cruz (sc-515120)
Rabbit anti-LHR	1:150	Santa Cruz (sc-25828)
Rabbit anti-SF-1	1:150	Abcam (ab65815)
Rabbit anti-StAR	1:100	GeneTex (GTX105716)
Rabbit anti-17β-HSD	1:150	GeneTex (GTX114480)
Rabbit anti-CYP11A1	1:200	GeneTex (GTX56293)
Rabbit anti-CYP17A1	1:200	GeneTex (GTX56294)
Goat anti-CYP11A1	1:50	Santa Cruz (sc-180143)
Rabbit anti-CD271	1:100	Promega (G3231)
Rabbit anti-Nestin	1:100	Millipore (ABD69)
Mouse anti-Nestin	1:100	Arigo (arg52345)
Rabbit anti-PDGFRα	1:200	Abcam (ab203491)
Rabbit anti-CONNEXIN 43	1:500	CST (3512s)
Rabbit anti-TOM 20	1:200	Santa Cruz (sc-11415)
Rabbit anti-Caspase 3	1:100	CST (9662s)
Rabbit anti-Ki67	1:100	Abcam (ab15580)
Rat anti-α-tubulin	1:100	Bio-Rad (MCA78G)
Rabbit anti-PDGFRβ	1:200	CST (3169s)
Rabbit anti-CD31	1:100	ARIGO (ARG52748)
Mouse anti-NG2	1:100	Sigma (MAB5384)
Rabbit anti-FSHR	1:100	Abcam (ab113421)
Rabbit anti- DDX4	1:100	CST (8761s)
Mouse anti Vimentin	1:100	Abcam (92547)
α-SMA	1:500	Abcam (7817)

Goat Anti-rabbit IgG Alexa 488	1:1000	Invitrogen (A-11034)
Goat Anti-rabbit IgG Alexa 594	1:1000	Invitrogen (A-11037)
Goat Anti-rabbit IgG Alexa 647	1:1000	Invitrogen (A-21244)
Goat Anti-mouse IgG Alexa 488	1:1000	Invitrogen (A-11001)
Goat Anti-mouse IgG Alexa 594	1:1000	Invitrogen (A-11032)
CD140a-PE	1:50	BD (556002)
Annexin V-PI	1:50	BD (565547)
CD90-APC	1:50	Cat# 559869
CD105-FITC	1:50	Cat# 561443
CD73-PE	1:50	Cat# 550257
CD34-FITC	1:50	Cat# 555821
CD45-FITC	1:50	Cat# 555482

Supplementary Table S4. Primers used in this study.

Gene	Forward Primer	Reverse Primer
GLI2	CTCACCTCCGTCAATGCCA	AGGCTCGGTCTTGACCTTG
PTCH2	CACATCCATCAACAACATGG	AAGCAGCAGAGCACATCGAG
LHR	CCTGACAAGTCGTTACAA	TATGAGCAGCAGATAGAGT
3β-HSD	AGGACAGTTCTATTACATCTC	TACATCAGGGCTAAAGGA
StAR	GGAGTGGAACCCTAATGT	ATCTCGTGAGTAATGAATGTATC
CYP11A1	ATCAATATGCTGGAGAACTT	CAGGATGAGGTTGAATGT
17β-HSD	AGGAGTGTGAAGATTATAC	TGATGTTACAATGGATGA
SF-1	AATGCCTACAGTTATTCC	GAGTCAGGTCTTCTATCT
COL2A	AGCAAGAGCAAGGAGAAGA	GGAGCCAGGTTGTCATCT
AGGRECAN	TACGATGTCTACTGCTATGT	GAAGGTGAACTGCTCAAG
PPARγ	CCATTCACAAGAACAGAT	CAGAATAATAAGGTGGAGAA
FABP4	GATCATCAGTGTGAATGG	TAAGGTTATGGTGCTCTT
ALP	CATAACATCAGGGACATTG	ATCTCATACTCCACATCAG
SPARC	AGAGGGATGAAGACAACA	GCTTCTCATTCTCATGGAT
RUNX2	ACCATAACCGTCTTCACAA	GAGGTCCATCTACTGTAACTT
GAPDH	CTCTGGTAAAGTGGATATTG	GGTGGAATCATACTGGAA

Supplementary Excel S1. Result of ADSCs，TSCs and TCs by RNA-seq.
